# Nanoparticle-mediated overexpression of RacGAP1 protects against renal ischemia/reperfusion injury by maintaining mitochondrial homeostasis

**DOI:** 10.1042/CS20256110

**Published:** 2025-11-27

**Authors:** Weiran Zhou, Shiqiang Tong, Jinbo Yu, Jun Chen, Yi Fang, Yuxin Nie, Yiqin Shi, Nana Song, Xuesen Cao, Xiaoqiang Ding, Shuan Zhao

**Affiliations:** 1 Divisionof Nephrology, Zhongshan Hospital, Fudan University, Shanghai, China; 2 Shanghai Key Laboratory of Kidney Diseases and Blood Purification, Shanghai, China; 3 Shanghai Institute of Kidney and Dialysis, Shanghai, China; 4 Shanghai Medical Center of Kidney, Shanghai, China; 5 Key Laboratory of Smart Drug Delivery, Ministry of Education, School of Pharmacy, Fudan University, Shanghai, China

**Keywords:** acute kidney injury, LCP, mitochondrial homeostasis, nanoparticles, RacGAP1

## Abstract

Acute kidney injury (AKI) is recognized as a critical clinical problem, and pharmacological therapeutic options for AKI remain limited. Our previous study confirmed that Rac GTPase-activating protein 1 (RacGAP1) effectively promoted the repair of tubular epithelial cells *in vitro*. Further investigation is needed to determine whether boosting the expression of RacGAP1 *in vivo* helps protect against AKI. Herein, lipid-coated calcium phosphate (LCP) nanoparticles loaded with RacGAP1 plasmids (pRacGAP1-LCP) were generated and subsequently characterized based on their size, zeta potential, and morphological features. Animal models of AKI induced by ischemia/reperfusion (I/R) injury (IRI) were established in C57BL/6 mice, and pRacGAP1-LCP was injected into the tail vein to explore the role of RacGAP1 on renal IRI *in vivo*. The therapeutic efficacy of pRacGAP1-LCP against IRI was assessed through western blotting, real-time PCR, and histological analyses. The effects of RacGAP1 on mitochondrial homeostasis were further examined in mouse renal tubular epithelial cells (mRTECs). Serial administrations of pRacGAP1-LCP led to a significant increase in RacGAP1 expression in murine kidneys. This therapeutic intervention effectively attenuated AKI, as evidenced by down-regulation of AKI biomarkers, amelioration of renal histopathological damage, and suppression of both apoptosis and inflammatory responses. Characteristic mitochondrial abnormalities, diminished ATP production, and excessive lipid droplet accumulation were observed in tubular cells of IRI mice. Notably, pRacGAP1-LCP treatment reversed these pathological alterations and up-regulated the expression of PGC-1α and CPT-1α, indicating that RacGAP1 exerted its reno-protective effects through enhanced mitochondrial biogenesis and fatty acid oxidation (FAO). To further investigate the role of RacGAP1 in mitochondrial homeostasis, we employed an ATP depletion-repletion (ATP D-R) model in mRTECs. Crucially, RacGAP1 effectively restored ATP production, mtDNA copy number, and oxygen consumption rate (OCR) in mRTECs after ATP D-R treatment. RacGAP1 overexpression also suppressed mitochondrial depolarization, fragmentation, and reactive oxygen species (ROS) generation. Conversely, RacGAP1 knockdown exacerbated mitochondrial defects in mRTECs exposed to ATP D-R. In summary, this study uncovers that RacGAP1 overexpression protects against renal injury and mitochondrial dysfunction, highlighting its therapeutic promise for AKI. The LCP nanoparticle exhibits potential as a precise and efficient delivery platform and presents a viable option for AKI therapy.

## Introduction

Acute kidney injury (AKI), characterized by an abrupt decline of kidney function, poses a global public health challenge with considerable mortality rates [1]. AKI encompasses a spectrum of etiologies from sepsis to nephrotoxin exposure. Among these, ischemia/reperfusion injury (IRI) is a predominant cause, particularly following cardiac surgery, renal transplantation, or hypovolemic shock. Despite its clinical significance, effective therapeutic strategies to promote renal recovery remain limited, underscoring an urgent need to elucidate novel molecular targets and therapeutic interventions [2].

Mitochondrial dysfunction is a fundamental mechanism underlying the pathogenesis of AKI [3]. Proximal tubular epithelial cells (PTECs) are metabolically active cells and are particularly vulnerable to ischemia and hypoxia due to their high energy demand and relatively low perfusion [4]. IRI significantly weakens oxidative phosphorylation (OXPHOS), disrupts the delicate balance of mitochondrial homeostasis, leading to impaired adenosine triphosphate (ATP) production, excessive reactive oxygen species (ROS) generation, and initiation of apoptotic pathways [5,6]. Central to this dysfunction is the disruption of mitochondrial biogenesis and a profound suppression of fatty acid oxidation (FAO), which is the primary energy source for PTECs [7]. Given the pivotal role of mitochondria in energy metabolism, exploring new targets maintaining mitochondrial homeostasis in PTECs is essential for AKI treatment.

Rac GTPase-Activating Protein 1 (RacGAP1), also known as MgcRacGAP, is a multifaceted protein implicated in cytokinesis, cell proliferation, cell migration, and signal transduction [8–12]. Our previous work demonstrated that RacGAP1 expression was significantly up-regulated in injured kidneys following IRI or cisplatin-induced nephrotoxicity. Functionally, RacGAP1 promoted proliferation and suppressed apoptosis *in vitro*, suggesting its facilitative role in the repair processes of tubular epithelial cells [13]. However, its specific function, protective mechanisms, and therapeutic potential in AKI remain unexplored.

Recent studies have reported that RacGAP1 was involved in the regulation of mitochondrial homeostasis, including mitochondrial biogenesis, mitochondrial dynamics, and mitophagy. Specifically, RacGAP1 promoted OXPHOS, increased ATP content, and up-regulated the expression of PGC-1α, a key mitochondrial biogenesis regulator in breast cancer cells [14]. Based on our preliminary investigations and existing literature demonstrating the involvement of RacGAP1 in the regulation of mitochondrial function, we hypothesize that RacGAP1 plays a crucial protective role in AKI by improving mitochondrial homeostasis. Furthermore, we speculate that up-regulating RacGAP1 expression in injured tubular epithelial cells during AKI may be a promising approach to attenuate damage.

Gene therapy for AKI holds immense potential, but conventional delivery methods face several significant challenges, including poor stability, low delivery efficiency, lack of organ specificity, and immunogenicity [15]. Hence, there is an urgent need to develop a safe and efficient method for gene delivery to treat AKI. Lipid-coated calcium phosphate (LCP) nanoparticles have emerged as a promising drug delivery platform due to their biocompatibility, biodegradability, and ability to encapsulate nucleic acids effectively [16–18]. However, there is still a lack of research on LCP nanoparticle-based gene therapy in the field of AKI. Therefore, we designed and synthesized LCP nanoparticles to deliver the RacGAP1 plasmids. To achieve kidney-specific targeting, the phosphatidylserine was incorporated into the nanoparticles because of its strong binding affinity to kidney injury molecule-1 (KIM-1), a serine receptor exhibiting heightened expression on damaged renal tubular epithelial cells [19]. Furthermore, serine-modified nanoparticles have been demonstrated to exhibit kidney-targeting properties [20]. We hypothesized that nanoparticle-mediated targeted delivery of RacGAP1 would contribute to mitochondrial homeostasis and confer a protective effect against AKI.

In this study, phosphatidylserine-modified LCP nanoparticles loaded with RacGAP1 plasmids (pRacGAP1-LCP) were synthesized as a novel gene delivery system in ischemic AKI. pRacGAP1-LCP could safely and efficiently deliver the RacGAP1 overexpression plasmids to the injured renal tubules and elevate RacGAP1 protein levels *in vivo*, and three-dose administration of pRacGAP1-LCP was sufficient to attenuate renal injury induced by IRI. pRacGAP1-LCP enhanced renal repair by promoting mitochondrial biogenesis and FAO, ultimately improving mitochondrial quality and mitigating renal damage. Our results highlight the translational potential of nanoparticle-based RacGAP1 gene therapy for ischemic AKI.

## Materials and methods

### Synthesis and characterization of LCP nanoparticles

LCP nanoparticles were synthesized using the water-in-oil reverse micro-emulsion method [21]. The oil phase was obtained by adding 6 ml of Igepal CO-520 (N828401, Macklin) to 14 ml of cyclohexane and stirring for 15 min. The calcium phase was prepared by adding 80 μg of plasmids to 300 μl of 2.5 M CaCl₂ solution, which was then dripped into the oil phase. 300 ml of 12.5 mM Na₂HPO₄ (pH>9) was dispersed in 20 ml oil phase, and after stirring, 1,2-dioleoyl-sn-glycero-3-phosphate (D864427, DOPA) (Macklin) (20 mg/ml, 50 μl) in chloroform was added and stirred further to form the phosphate phase. The calcium phase was subsequently merged with the phosphate phase, followed by the addition of DOPA to the combined solution for 45 min. Ethanol (40 ml) was added subsequently, and the mixture was centrifuged at 12500 *g* for 20 min. The supernatant was discarded, and the precipitate was washed with ethanol, followed by another centrifugation at 12500 *g* for 20 min. After three washes, the supernatant was discarded, and the precipitate was washed with ethanol three times. The precipitate represented the calcium phosphate core wrapped with the RacGAP1 plasmids.

For the lipid coating, 10 mg of the calcium phosphate core was mixed with 300 μl of 20 mM cholesterol (C8667,Sigma), 300 μl of 20 mM DOTAP(132172–61-3, AVT Pharmaceutical Tech Co,. Ltd.), 200 μl of 20 mM DSPE-PEG2000 (6434–3373-2K, Yarebio), and 70 μl of 20 mM 1,2-dipalmitoyl-sn-glycero-3-phospho-L-serine (145849–32-7, Avanti). The chloroform was then evaporated using rotary evaporation to form a thin lipid film. The film was hydrated with 5% glucose and sonicated to obtain LCP nanoparticles loaded with the RacGAP1 plasmids (pRacGAP1-LCP). Nanoparticles encapsulating the negative control plasmid (pNC-LCP) and DiR probe-labeled pRacGAP1-LCP (DiR-pRacGAP1-LCP) were prepared using an analogous approach.

The morphology and size of pNC-LCP and pRacGAP1-LCP were observed under a transmission electron microscope (Thermo Fisher Scientific) after negative staining with 1% phosphotungstic acid. The size distribution and the zeta potential were measured using the dynamic light scattering (DLS) detector (Malvern).

### Renal IRI models

Male C57BL/6 mice, aged eight weeks, were obtained from the Animal Center of Fudan University. The mice were fed a standard laboratory rodent diet (Product Name: Irradiated sterile experimental mouse growth and breeding feed, Catalogue Number: XTI01CR-010, Manufacturer: Jiangsu Synergy Medical Bioengineering Co., Ltd.). Mice were housed in a specific pathogen-free (SPF) facility under standardized conditions: a 12 h light/12 h dark cycle, with free access to food and water. All C57BL/6 mice were randomly assigned to the experimental groups.

The induction of bilateral IRI in mice was performed under general anesthesia. Renal pedicles were meticulously dissected for both kidneys. Nontraumatic microvascular clamps were then applied to simultaneously occlude blood flow to both kidneys for precisely 21 min, with the mice core body temperature maintaining at 37°C. Following the ischemic interval, the clamps were removed to initiate reperfusion. Mice in the sham group underwent the same surgical procedures but without renal pedicle clamping. The mice were sacrificed 72 h post-reperfusion, and the kidneys were obtained for further analysis. For biosafety evaluation, the liver and spleen were also excised for further histological analysis. The anesthetic agent was isoflurane, and the mice were euthanized through cervical dislocation.

### Biodistribution of LCP nanoparticles

Previous reports have demonstrated that the glomerular filtration barrier underwent significant structural changes and had increased permeability in the injured kidney, enabling the passage of nanoparticles [22,23]. Consequently, the bio-distribution of nanoparticles was examined in mice models of IRI rather than in normal kidneys. Bilateral renal pedicles were clamped for 21 min, and the mice were subsequently divided into two groups. Immediately after reperfusion, one group received an injection of DiR-pRacGAP1-LCP through the tail vein, while the other group received an injection of free DiR. The mice were euthanized at 2, 6, 12, 24, and 48 h post-injection. Hearts, livers, spleens, lungs, and kidney tissues were harvested, and the organ surfaces were cleaned with physiological saline and dried.

To assess the accumulation of DiR-pRacGAP1-LCP in various organs, a PerkinElmer IVIS Spectrum small-animal imaging system was utilized. The near-infrared fluorescent image parameters were preset using DiR dye, with the excitation wavelength at 748 nm and the emission wavelength at 780 nm.

### Experimental design for assessing the renal protective efficacy of pRacGAP1-LCP

C57BL/6 mice subjected to renal IRI were randomly allocated to three groups (*n* = 5 each). The groups received tail vein injections of 100 μl saline, 100 μl containing 40 μg of pNC-LCP, or 100 μl containing 40 μg of pRacGAP1-LCP, respectively. These injections were administered three times: immediately after reperfusion, 24 h post-reperfusion, and 48 h post-reperfusion. Blood and kidney tissues were collected from all groups 72 h after reperfusion.

### Histology staining

Renal tissues were sliced into 4μm-thick sections and stained with hematoxylin and eosin (H&E) staining kit (C0105S, Beyotime) following standard procedures. To ensure objective and accurate assessment, a professional pathologist, who was blinded to the experimental groups, performed the histological evaluation. For each tissue section, ten randomly selected fields were examined. Each field was scored based on the degree of tissue damage according to the following criteria: 0, no damage; 1, < 25% damage; 2, 25–50% damage; 3, 50–75% damage; 4, > 75% damage [24]. The scores from the ten fields per sample were averaged to obtain a mean histopathological injury score for each mouse. The liver and spleen were also fixed in 10% formalin, embedded in paraffin, sectioned, and stained with H&E for the histological analysis.

## Immunohistochemistry（IHC)

IHC assay was performed as previously described [25]. Briefly, we prepared tissue sections through fixation and paraffin embedding, followed by deparaffinization and rehydration. Antigen retrieval was then performed using heat treatment to expose epitopes. Then, sections were incubated with hydrogen peroxide to block endogenous peroxidase activity and nonspecific binding. Primary antibodies targeting the following proteins were applied and allowed to bind overnight at 4°C: RacGAP1 (1:1000, GTX113320, GeneTex), KIM-1 (1:100, TA322411, Origene), cleaved caspase-3 (1:400, 9664, CST, 1:400), F4/80 (1:400, 70076, CST), CPT-1α (1:200, ab234111, Abcam), and PGC-1α (1:200, ab191838, Abcam). After washing, a secondary antibody conjugated to an enzyme such as horseradish peroxidase (HRP) (1:2000, ab205718, Abcam) is added at 37°C for 30 min. The localization of peroxidase was visualized using a DAB kit (GK500710, Gene Tech), and tissue sections were examined under a light microscope (Leica).

The IHC scoring method involved randomly selecting 10 representative images per tissue sample, analyzing them with ImageJ (version 1.25), and then calculating the average value. To ensure the specificity of IHC staining, negative controls were included in every experiment. The primary antibody was replaced with an antibody diluent. This control was processed identically to the test slides throughout the entire protocol.

### Terminal deoxynucleotidyl transferase-mediated dUTP nick-end labeling (TUNEL) staining

To assess the presence of apoptotic cells, TUNEL staining was performed according to the manufacturer’s instructions (C1086, Beyotime Biotechnology). First, the slides were deparaffinized and rehydrated. Then, the slides were incubated with a proteinase K (20 mg/ml) for 15 min to permeabilize the cells, and then endogenous peroxidase activity was quenched with hydrogen peroxide. The TUNEL reaction mixture, containing the TdT enzyme and labeled nucleotides, was applied for 1 h and the sections were incubated in a humidified dark chamber. DAPI was used to label the nuclei, and apoptotic cells were defined as green fluorescent cells. From each slide, ten fields were randomly chosen for quantitative analysis, focusing on the number of TUNEL-positive cells across four different groups. TUNEL positive cells are quantified using ImageJ (version 1.25) as previously described [26].

### Transmission electron microscopy for observing mitochondrial morphology

Electron microscopic analysis was conducted following a previously established protocol [27]. Ultrathin sections were meticulously cut and then post-stained with uranyl acetate (19481, TED PELLA) for 10 min, followed by a 5-min staining with lead citrate (15326, Sigma). This staining process enhanced the contrast and clarity of the cellular structures within the sections. Finally, the stained sections were observed under a transmission electron microscope (Thermo Fisher Scientific) operating at 120 kV for proximal tubules. Proximal tubules were then examined at low magnification (×2,500) as well as high magnification (×7,500) images. Mitochondria morphology and the amount of lipid droplets per field were counted in 10 randomly selected fields for each mouse. The length of mitochondria was calculated by ImageJ (version 1.25). The number of mitochondria per field was estimated as previously described [28].

A transmission electron microscope was also applied to observe mitochondrial morphology of mRTECs. The culture medium was aspirated and the cells were washed gently with phosphate-buffered saline (PBS). The adherent cells were then detached using a trypsin-EDTA solution. The resulting cell suspension was transferred to a centrifuge tube and pelleted by centrifugation at 1000 rpm for 5 min. The supernatant was carefully discarded, and the pellet was gently resuspended in a 2.5% Gluta fixative (G1102, Servicebio). Following primary fixation, the fixed cell pellet was stored in the fixative at 4°C for electron microscopy. Mitochondria morphology, the mitochondria perimeter, and the numbers of autophagosomes were analyzed in ten randomly selected cells for each group using ImageJ (version 1.25).

### ATP assay

The ATP concentration was measured using an ATP determination kit (119107, Sigma) according to the manufacturer’s protocol. Briefly, kidney samples were homogenized in the provided assay buffer and subsequently filtered through a 10  kDa molecular weight cut-off (MWCO) spin filter (FUF051, Beyotime) for protein removal. The resulting filtrates were then mixed with ATP probe, followed by incubation at room temperature for 30 min protected from light. Absorbance was measured at 570  nm using a microplate reader, and ATP concentrations were determined based on a standard curve. The ATP content was ultimately expressed as nmol ATP/mg protein.

For cell experiments, we also used the same ATP determination kit with the similar protocol. Cells were lysed using lysis buffer. The lysate was then centrifuged, and the resulting supernatant was collected for ATP quantification. The ATP content was expressed as nmol ATP/mg protein.

### Oil red O staining

To evaluate renal lipid accumulation, frozen kidney tissue sections were stained with Oil Red O (O0625, Sigma) solution as previously described [29]. The staining was performed by incubating the sections in Oil Red O in the dark for 15 min. After several washes, 60% isopropanol was applied to enhance the dissolution and specificity of the dye. The sections were then counterstained with hematoxylin for 10 min. All samples were examined under a light microscope (Leica). Ten randomly chosen fields were evaluated for each mouse kidney tissue section, and an average score was calculated.

### Cell culture and ATP depletion and repletion (ATP D-R) model

mRTEC line (CP-M062, Pronosai Biotechnology Co., Ltd.) were cultured in medium (RPMI 1640, Gibco) containing 10% fetal bovine serum (FBS, A5256701, Gibco) , under a humidified atmosphere of 5% CO₂ at 37°C. When the cultures reached 80% fusion, 0.25% trypsin/EDTA digestion was performed for passaging.

To establish the ATP depletion-reperfusion (ATP D-R) model *in vitro*, the mRTECs were then incubated with 10 μM carbonyl cyanide m-chlorophenylhydrazone (CCCP, HY-100941, MCE) in medium without FBS for 4 h, followed by a 2-h recovery period in a complete culture medium. Dimethyl sulfoxide (DMSO, HY-Y0320, MCE) was used as the control.

### Overexpression and interference lentiviruses of RacGAP1

The overexpression and interference lentiviruses of RacGAP1 as well as the control lentiviruses were synthesized by Shanghai Tsingke Biotechnology Co., Ltd. and transfected into the cells according to the manufacturer’s instructions [30]. When the cells reached about 70% confluency, they were infected with lentiviruses with 5 μg/ml polybrene (TR-1003, Merck) and the optimal multiplicity of infection (MOI) was 10. After 8 h, the medium was replaced with fresh complete medium. Infection efficiency was monitored at 72 h post-infection using the luciferase reporter gene. The transduced cells were selected with puromycin (Gibco, A1113803) at a concentration of 1 μg/ml. Selection was continued until no cell death was observed in the puromycin-containing medium.

### Determination of ROS

MitoSOX Red staining was performed to detect mitochondrial ROS production [31]. Cells were grown on clear bottom plates. After treatments, cells were incubated with MitoSOX Red reagent (5 μM) for 30 min at 37°C in the dark. Following incubation, cells were washed thoroughly to remove unbound dye. Nuclei were stained with Hoechst 33,342 (HY-15559, MCE) for 5 min. Stained cells were examined using an Olympus FV1000 confocal microscope for analysis.

Quantitative analysis was conducted by measuring the fluorescence intensity with cells grown on 96-well black/clear bottom plates. Fluorescence intensity was immediately measured using a microplate reader with excitation/emission wavelengths set at 510/580 nm. The obtained fluorescence values were normalized to cell number for accurate quantification.

### JC-1 staining

The mitochondrial membrane potential was evaluated via a JC-1 staining kit (C2003S, Beyotime Biotechnology). Concisely, cells were rinsed twice with PBS and subsequently incubated with 500  μl of JC-1 staining solution in each well for 20 min in the dark. After washing with JC-1 assay buffer, stained cells were examined using an Olympus FV1000 confocal microscope.

We analyzed the JC-1 fluorescence intensity with the wavelength-based approach instead of the pseudocolor. Specifically, the fluorescence intensities of JC-1 were measured at 530 nm (green fluorescence, monomeric form) and 590 nm (red fluorescence, J-aggregate form) using a fluorescence spectrophotometer (Thermo Fisher Scientific). The ratio of fluorescence intensity at 590 nm to that at 530 nm was calculated to assess mitochondrial membrane potential. Furthermore, we have included a new positive control group (cells treated with 50 μM CCCP for 30 min and without ATP reperfusion) to validate the specificity of JC-1 staining.

### Mitochondrial stress assay

The Seahorse XF-96 Extracellular Flux Analyzer (Agilent) was employed to measure the oxygen consumption rate (OCR). Briefly, mRTECs were initially cultured in the XF96 cell culture microplate (102601, Agilent). Before the assay, the growth medium was removed and cells were washed with serum-free Seahorse DMEM medium. Then, 180 µl of Seahorse DMEM medium supplemented with 2 mM glutamine, 1 mM pyruvate, and 10 mM glucose was added to each well. Cells were incubated for 45–60 min to allow temperature and pH equilibration. Mitochondrial function was assessed using the XF Cell Mito Stress Test Kit (103015, Agilent) through sequential injections of 1.5 µM oligomycin, 1 µM carbonyl cyanide 4-(trifluoromethoxy)phenylhydrazone (FCCP), and 0.5 µM rotenone/antimycin A. OCR was recorded in real-time following the injection of each compound.

Following the Seahorse assay, cell number was quantified for normalization by staining nuclei with Hoechst 33,342 for 10 min at 37°C protected from light. The cell number was obtained based on nuclear counts performed immediately following the assay. The OCR values were then standardized against cell count and expressed as pmol/min/10⁴ cells to enable direct comparison across groups. All standard Seahorse parameters, including basal respiration, maximal respiration, ATP production, and spare respiratory capacity, were analyzed with *P* values indicated.

### Western blot analysis

Proteins were extracted from cultured cells or mouse kidneys using RIPA lysis buffer (P0013B, Beyotime Biotechnology), which was supplemented with PMSF (ST506, Beyotime Biotechnology). Extracted proteins were separated via SDS-PAGE and transferred onto PVDF membranes (IPVH00010, Millipore). Membranes were blocked with 5% milk and incubated overnight at 4°C with primary antibodies targeting the following proteins: RacGAP1 (1:1000, GTX113320, GeneTex), KIM-1 (1:1000, AF1817, R&D Systems), NGAL (1:1000, ab125075, Abcam), Bax (1:1000, 2772T, CST), Bcl-2 (1:1000, 15071, CST), Cytochrome c (1:1000, ab13575, Abcam), cleaved caspase-3 (1:1000, 9664, CST), PGC-1α (1:1000, ab191838, Abcam), CPT-1α (1:1000, ab234111, Abcam), PINK1 (1:1000, 6946, CST), Parkin (1:1000, 4211, CST), LC3B (1:1000, 2775, CST), MFN-2 (1:1000, 9482, CST), OPA-1 (1:1000, 80471, CST), and β-actin (1:2000, GTX110003, GeneTex). Before incubation with secondary antibodies (1:2000, Jackson ImmunoResearch Inc.), the membranes were thoroughly washed with TBST. Visualization of the target protein bands was achieved using the LAS-3000 detection system, and the signal intensity was quantified with the assistance of ImageJ (version 1.25).

### Quantitative real-time PCR

Total RNA was extracted using the Trizol reagent (Invitrogen). Reverse transcription and real-time PCR were performed using PrimeScript RT Master Mix and SYBR Premix ExTaqTM (TaKaRa) on a 7500 real-time PCR system (Thermo Fisher Scientific). The relative quantification was calculated as 2^−ΔΔCt^. The following primers (Sangon) 5′ to 3′ were used:

Mouse RacGAP1-F: AGAGTCCAGACACTAAGATG

Mouse RacGAP1-R: TTACTTGAGGTACGGAGCAG

Mouse KIM-1-F: AGGCGCTGTGGATTCTTATG

Mouse KIM-1-R: AAGCAGAAGATGGGCATTGC

Mouse NGAL-F: TGGCCCTGAGTGTCATGTG

Mouse NGAL-R: CTCTTGTAGCTCATAGATGGTGC

Mouse TNF-α-F: CAGACCCTCACACTCACAAACCAC

Mouse TNF-α-R: CCTTGTCCCTTGAAGAGAACCTG

Mouse IL-6-F: TAGTCCTTCCTACCCCAATTTCC

Mouse IL-6-R: TTGGTCCTTAGCCACTCCTTC

Mouse LCAD-F: TCACCAACCGTGAAGCTCGA 

Mouse LCAD-R: CCAAAAAGAGGCTAATGCCATG

Mouse MCAD-F: TAACATACTCGTCACCCTTC 

Mouse MCAD-R: ATGCCTGTGATTCTTGCT

Mouse CPT-1α-F: CCAGGCTACAGTGGGACATT 

Mouse CPT-1α-R: GAACTTGCCCATGTCCTTGT

Mouse NRF-2-F: GATTCACGCATAGGAGCACTG 

Mouse NRF-2-R: CTTCCATTTACGGAGACCCAC

Mouse PPAR-γ-F: TCCGTAGAAGCCGTGCAAGA 

Mouse PPAR-γ-R: CAGCAGGTTGTCTTGGATGTC

Mouse TFAM-F: CTTCCCAAGACTTCATTTC 

Mouse TFAM-R: AAGACCTCGGTCAGCATA

Mouse PGC-1α-F: CTCCATGCCTGACGGCACCC 

Mouse PGC-1α-R: GCAGGGACGTCTTTGTGGCT

Mouse Actin-F: AGCCATGTACGTAGCCATCC

Mouse Actin-R: GCTGTGGTGGTGAAGCTGTA

### Quantification of mitochondrial DNA (mtDNA) content

Total DNA was extracted using the DNeasy® Blood & Tissue Kit (Qiagen, 69504). mtDNA was quantified as previously described [32]. Two sets of PCR primers—specific to the mitochondrial NADH dehydrogenase 1 (mt-ND1) gene and the β2-microglobulin gene (B2m) gene—were used to amplify mtDNA and nuclear DNA (nDNA), respectively. mtDNA copy number was determined by RT-qPCR. The relative mtDNA copy number, normalized to nDNA, was calculated using the 2⁻^ΔΔCT^ method.

Primer sequences 5′ to 3′ were as follows:

Mouse mt-ND1-F: CCCAACACCTCTTTACAGTG

Mouse mt-ND1-R: TAGAAGAGCGATGGTGAGAG

Mouse B2m-F: TGCTGTCTCCATGTTTGATGTATCT

Mouse B2m-R: TCTCTGCTCCCCACCTCTAAGT

### Statistical analysis

Data are presented as box plots showing the median and interquartile range as well as all individual data points, with whiskers defining the minimum and maximum values.

All data were firstly assessed for normality with the Shapiro–Wilk test and variance homogeneity with the Brown-Forsythe test. The biodistribution of LCP nanoparticles and *in vitro* studies were analyzed by two-way ANOVA. When the interaction was significant, the *P* values shown were derived from Sidak’s multiple comparisons test. When the interaction was not significant, the *P* values shown are from the main effects of the two-way ANOVA. Effect sizes are reported as partial η² (ηp²) for the experiments where *n* = 4 per group. For other *in vivo* data, one-way ANOVA was used for data satisfying both normality and homogeneity of variances, whereas Welch’s ANOVA was used for data that were normally distributed but had unequal variances. When a significant main effect was found in the ANOVA models (*P*<0.05), *post hoc* comparisons were conducted using Tukey’s test for ordinary ANOVA and the Games-Howell test for Welch’s ANOVA.

Statistical analyses were performed using GraphPad Prism version 9.5.1. A *P* value < 0.05 was considered to be statistically significant. The exact *P* values were labeled in all figures.

## Results

### The characterization and bio-distribution of pRacGAP1-LCP

**Figure 1:**
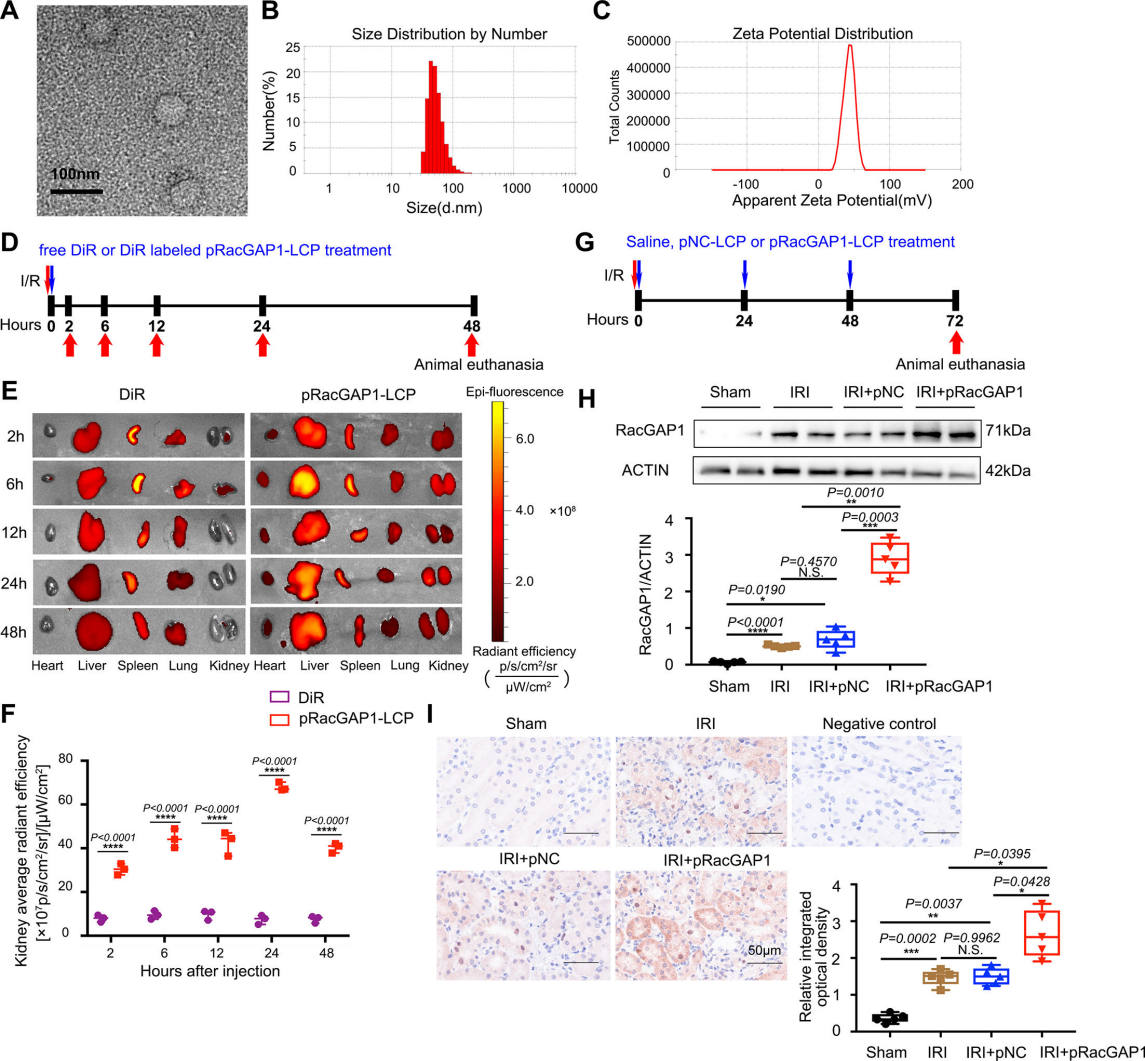
The characterization of pRacGAP1-LCP and the activation of RacGAP1 in kidney tubules from IRI mice. (**A**) Transmission electron microscope image of pRacGAP1-LCP. Scale bar = 100 nm. (**B**) Size distribution of pRacGAP1-LCP measured by DLS. (**C**) Zeta potentials of pRacGAP1-LCP measured by DLS. (**D**) Imaging schedule for evaluating the targeting property of nanoparticles *in vivo*. The mice were intravenously administered with DiR-labeled pRacGAP1-LCP and free DiR. To obtain detailed information on the targeting efficiency, mice were sacrificed, and the organs were collected and imaged at different time points. (**E**) Distribution of free Di-R and DiR-labeled pRacGAP1-LCP among organs at 2, 6, 12, 24, and 48 h after intravenous injection (*N* = 3). (**F**) Qualitative analyses of the fluorescent intensity of DiR-labeled pRacGAP1-LCP in kidneys, with significance analyzed using two-way ANOVA followed by the Sidak’s tests (*n* = 3). (**G**) Experimental design for animal studies. The groups received tail vein injections consisting of either saline, pNC-LCP, or pRacGAP1-LCP. These injections were administered three times: immediately following reperfusion, 24 h after reperfusion, and 48 h after reperfusion. Tissues were collected 72 h post-reperfusion. (**H**) Western blot and (**I**) IHC staining from mice of different groups showing the induction of RacGAP1 expression in the kidney tubule after pRacGAP1-LCP injection (*n* = 5). Scale bar = 50 μm. For each mouse, an average staining score was derived from ten random fields. Statistical analysis was performed with Welch’s one-way ANOVA followed by the Games-Howell post-hoc test. Data were presented as box plots showing the median and interquartile range, with whiskers extending from the minimum to the maximum value. *P<0.05; **P<0.01; ***P<0.001; ****P<0.0001; N.S., no significance.

Under the transmission electron microscope, pRacGAP1-LCP exhibited spherical morphology (Figure 1A). The average diameter was 123.4 nm ± 18.7 nm as determined by dynamic light scattering analysis, and the zeta potential was 42.7 mV (Figure 1B–C). The bio-distribution of pRacGAP1-LCP was studied in IRI mice via the spectrum optical imaging device. Following the establishment of the IRI model, mice were intravenously injected with pRacGAP1-LCP labeled with DiR or free DiR. The near-infrared fluorescence of kidneys was monitored at 2, 6, 12, 24, and 48 h post-injection (Figure 1D). Compared with the free DiR group, the pRacGAP1-LCP group exhibited significantly higher fluorescence intensity in the kidneys within 48 h after intravenous injection (Figure 1E). The maximum fluorescence intensity in the IRI kidneys was observed at 24 h post-injection, and the fluorescence remained detectable in the IRI kidneys even at 48 h after injection (Figure 1F). These results demonstrated the targeted delivery efficiency of pRacGAP1-LCP to the IRI kidney.

### pRacGAP1-LCP further up-regulated RacGAP1 in renal tubular cells in IRI mice

To further augment the transfection efficiency, pRacGAP1-LCP was injected intravenously three times in the subsequent study, specifically at 0 h, 24 h and 48 h post-reperfusion (Figure 1G). For comparison, nanoparticles encapsulating the negative control plasmids (pNC-LCP) were used to treat C57BL/6 mice after I/R surgery. Western blot results revealed a significant elevation in RacGAP1 protein levels in the kidneys of mice that received pRacGAP1-LCP injection (Figure 1H). Immunohistochemistry (IHC) staining demonstrated faint RacGAP1 expression in kidney tissues of the sham group and enhanced RacGAP1 expression in the tubular cells of mice subjected to IRI. Notably, the abundance of RacGAP1 further increased in the kidneys of mice treated with pRacGAP1-LCP, and its presence was detected in both the nucleus and cytoplasm (Figure 1I). These findings corroborated that plasmid-loaded nanoparticles effectively up-regulated the expression of RacGAP1.

### pRacGAP1-LCP protected against renal IRI in mice

**Figure 2:**
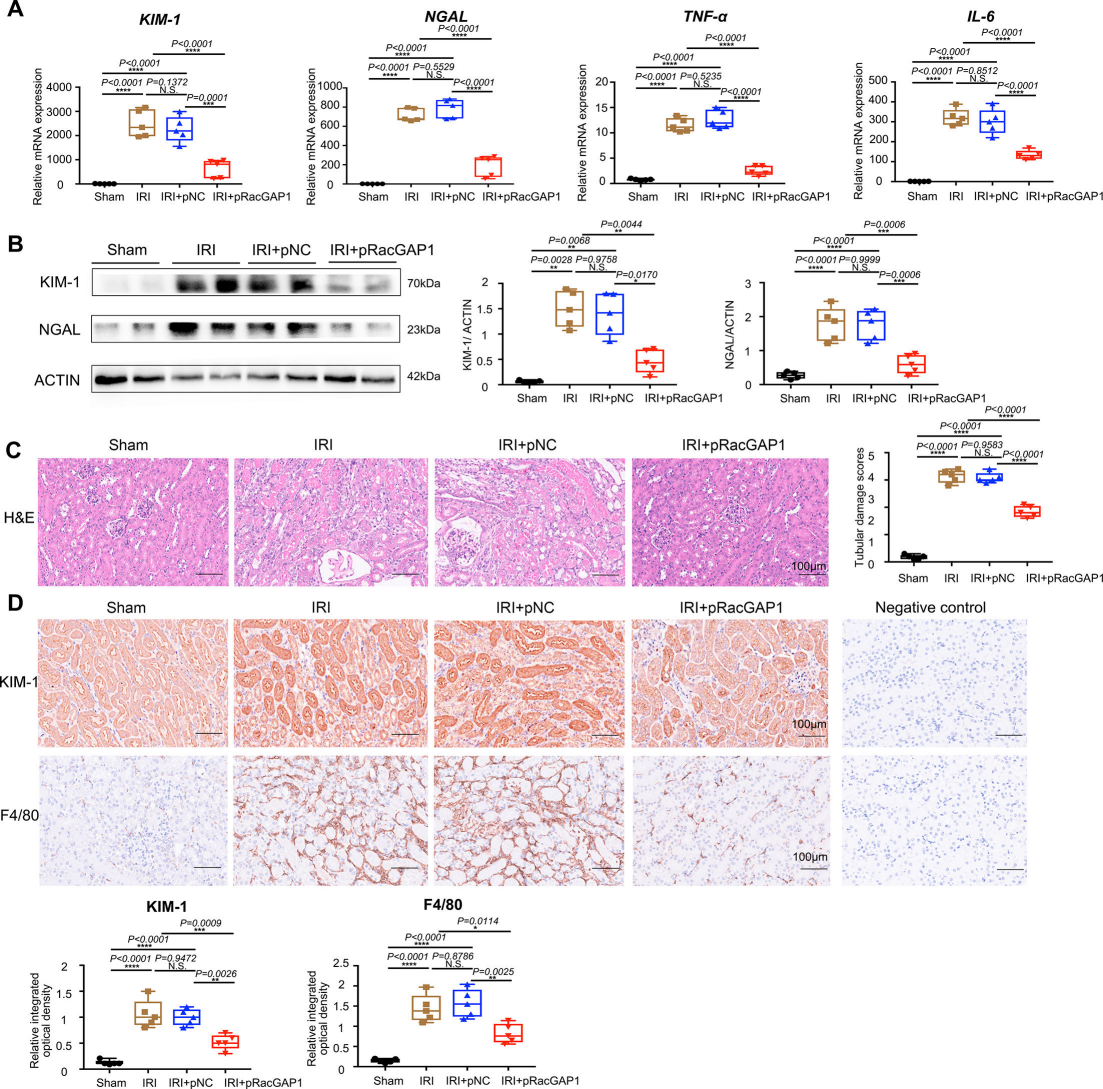
pRacGAP1-LCP ameliorated IRI-induced kidney injury. (**A**) Real-time PCR analysis showing the mRNA abundance for KIM-1, NGAL, TNF-α, and IL-6 in the kidney of Sham-, IRI-, IRI, and pNC-LCP-, or IRI and pRacGAP1-LCP -treated mice (*n* = 5). Statistical analysis was calculated with one-way ANOVA followed by Tukey’s test. (**B**) The protein levels of KIM-1 and NGAL in the kidneys of mice from different groups (*N* = 5). The protein levels of KIM-1 was analyzed with Welch’s one-way ANOVA followed by Games-Howell post-hoc test due to unequal variances. The protein levels of NGAL was analyzed by one-way ANOVA with Tukey’s test. (**C**) Representative micrographs of H&E staining and statistical analysis results, with significance analyzed using one-way ANOVA followed by Tukey’s tests (*n* = 5). For each mouse, an average staining score was derived from ten random fields. Scale bar = 100 μm. (**D**) Representative IHC staining and statistical analysis results for KIM-1 and F4/80, with significance analyzed using one-way ANOVA followed by Tukey’s test (*n* = 5). For each mouse, an average staining score was derived from ten random fields. Scale bar = 100 μm. Data were presented as box plots showing the median and interquartile range, with whiskers extending from the minimum to the maximum value. *P<0.05; **P<0.01; ***P<0.001; ****P<0.0001; N.S., no significance.

Considering that the nanoparticles could accumulate in the kidney and have induced RacGAP1 overexpression, we intended to evaluate the therapeutic effects of the nanoparticle on renal IRI. As illustrated in Figure 2A, the mRNA expressions of the tubular injury markers KIM-1 and neutrophil gelatinase-associated lipocalin (NGAL) in kidneys of pRacGAP1-LCP treated mice were notably reduced compared with those treated with pNC-LCP (Figure 2A). Additionally, the inflammatory response was observed in the kidneys. Real-time PCR results demonstrated a significant decrease in the abundance of TNF-α and IL-6 mRNA in the kidneys of mice subjected to pRacGAP1-LCP treatment (Figure 2A). In accordance with the PCR findings, pRacGAP1-LCP treatment also down-regulated the protein expression of KIM-1 and NGAL, as shown in Figure 2B. Furthermore, an analysis of hematoxylin and eosin (H&E)-stained kidney sections from each group revealed that pRacGAP1-LCP treatment significantly reduced tubular injury, with well-preserved brush borders in the proximal tubules (Figure 2C). Similarly, IHC results indicated that pRacGAP1-LCP treatment largely suppressed the expression of KIM-1 and F4/80, suggesting that RacGAP1 protected tubular cells from damage and inflammation (Figure 2D). Overall, these results imply that the overexpression of RacGAP1 alleviates IRI-induced tubular injury.

### pRacGAP1-LCP attenuated IRI-induced tubular cell apoptosis in mice

**Figure 3:**
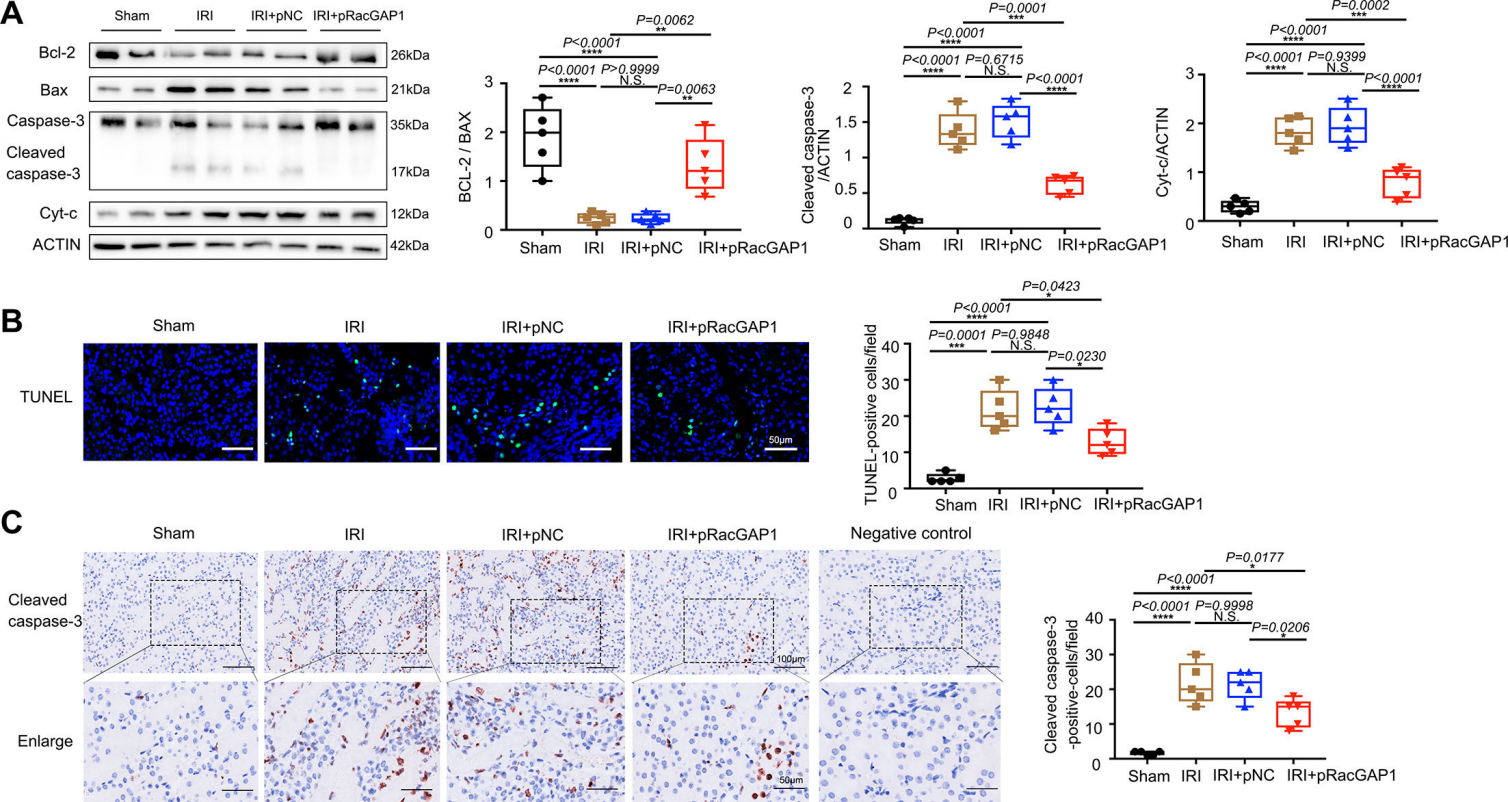
pRacGAP1-LCP attenuated IRI-induced tubular cell apoptosis. (**A**) Western blot assay showing the abundance of Bcl-2, Bax, cleaved caspase-3 and Cyt-c in the kidneys of mice from different groups (*n* = 5). Statistical analysis was calculated using one-way ANOVA followed by Tukey’s test. (**B**) Representative micrographs showing TUNEL staining and quantification (*n* = 5). For each mouse, an average staining score was derived from ten random fields. Significance was analyzed using one-way ANOVA followed by Tukey’s test. Scale bar = 50 μm. (**C**) Representative micrographs showing anti-cleaved caspase-3 IHC staining and quantification (*n* = 5). For each mouse, an average staining score was derived from ten random fields. Significance was analyzed using one-way ANOVA followed by Tukey’s test. Low-magnification image, scale bar = 100 μm. High-magnification image, scale bar = 50 μm. Data were presented as box plots showing the median and interquartile range, with whiskers extending from the minimum to the maximum value. *P<0.05; **P<0.01; ***P<0.001; ****P<0.0001; N.S., no significance.

As illustrated in Figure 3A, the injection of pRacGAP1-LCP significantly decreased the levels of Bax protein and increased the expression of Bcl-2 protein in the kidneys of IRI mice. Furthermore, the overexpression of RacGAP1 also resulted in a downregulation of cleaved caspase-3 and cytochrome c (cyt-c) expression in ischemic kidneys. To investigate cellular apoptosis in these kidney tissues, we employed TUNEL staining and IHC staining. Minimal TUNEL-positive staining was observed in the kidney tissues of sham-operated mice. Following renal IRI, a significant reduction in both TUNEL-positive cells was evident in the kidneys of mice treated with pRacGAP1-LCP, compared with those treated with pNC-LCP (Figure 3B). IHC assay also demonstrated that pRacGAP1-LCP treatment down-regulated the protein expression of cleaved caspase-3, as shown in Figure 3C. Collectively, these findings indicate that RacGAP1 exerts a protective effect against apoptosis in the kidneys of IRI mice.

### pRacGAP1-LCP promoted mitochondrial homeostasis and FAO during IRI

**Figure 4:**
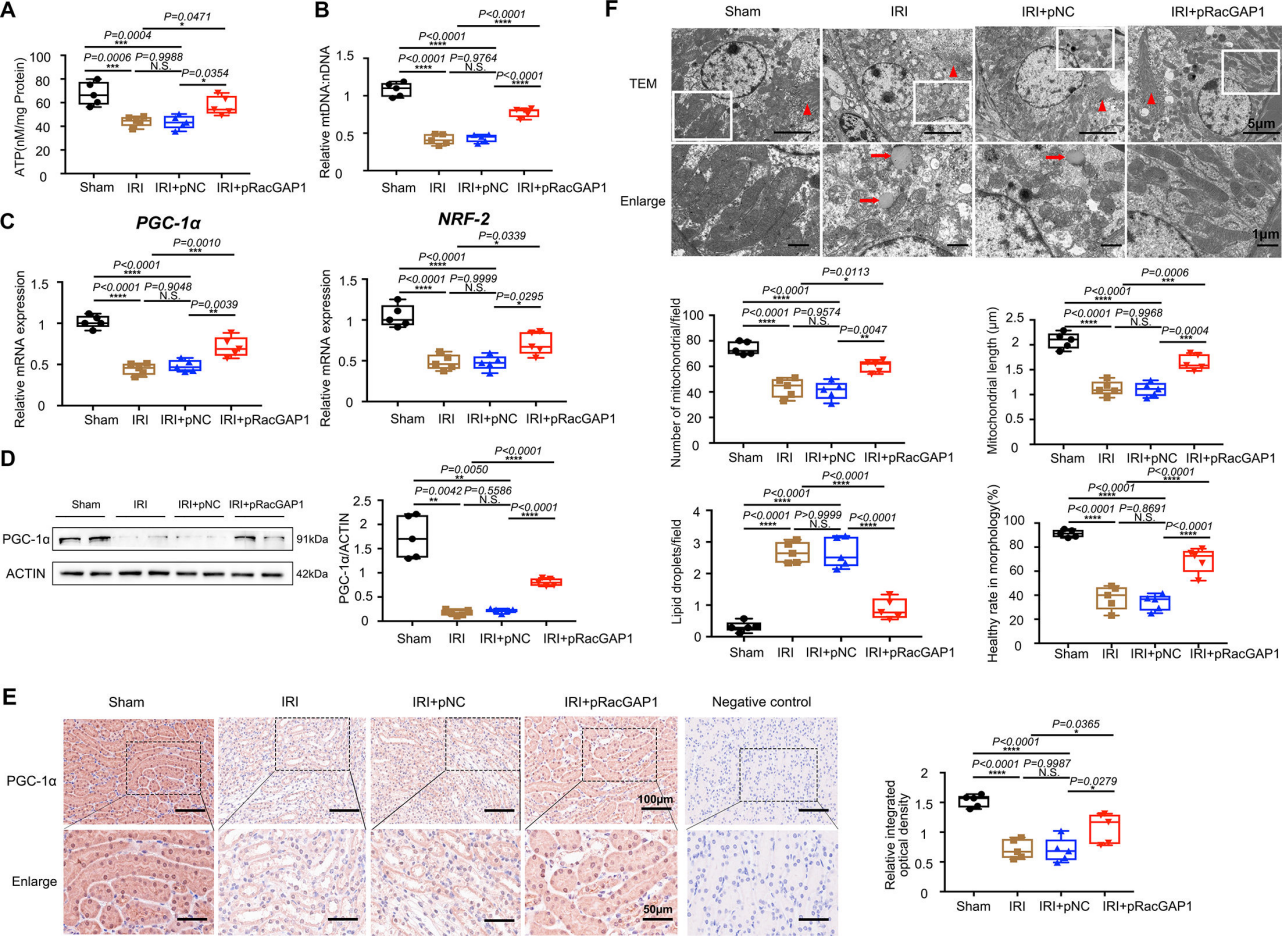
pRacGAP1-LCP protected against mitochondrial defect in kidney tubules in IRI mice. (**A**) The ATP levels in the kidney of mice from different groups (*n* = 5). Significance was analyzed using one-way ANOVA followed by Tukey’s test. (**B**) Real-time PCR analysis showing the mtDNA abundance (*n* = 5). Significance was analyzed using one-way ANOVA followed by Tukey’s test. (**C**) Real-time PCR analysis showing the mRNA abundance of PGC-1α and NRF-2 (*n* = 5). Significance was analyzed using one-way ANOVA followed by Tukey’s test. (**D**) Western blot assay showing the protein abundance of PGC-1α (*n* = 5). Statistical analysis was calculated with Welch’s one-way ANOVA followed by the Games–Howell *post hoc* test. (**E**) Representative IHC staining and statistical analysis results for PGC-1α with significance analyzed using one-way ANOVA followed by Tukey’s test (*n* = 5). For each mouse, an average staining score was derived from ten random fields. (**F**) Representative transmission electron microscopy images evaluating the mitochondrial morphology in proximal tubules of mice kidneys (*n* = 5). Red arrows denoted the lipid droplets. Red arrowheads indicated the brush border as the characteristic of proximal renal tubule epithelial cells. Low-magnification image, scale bar = 5 μm. High-magnification image, scale bar = 1 μm. For each mouse, ten proximal renal tubule epithelial cells were randomly imaged, with mitochondrial measurements averaged to provide one value per mouse. Quantification of the number of mitochondria, the mitochondria length, the healthy rate of mitochondria morphology and the number of lipid droplets were presented. Significance was analyzed using one-way ANOVA followed by Tukey’s test. Data were presented as box plots showing the median and interquartile range, with whiskers extending from the minimum to the maximum value. *P<0.05; **P<0.01; ***P<0.001; ****P<0.0001; N.S., no significance.

The maintenance of mitochondrial homeostasis is crucial for preserving the physiological function and survival of tubular cells [33]. To evaluate the role of RacGAP1 on mitochondrial homeostasis in tubular cells, ATP content analysis was performed. The results suggested that RacGAP1 enhanced ATP production in the kidneys of IRI mice (Figure 4A). PGC-1α and NRF-2 are both key regulators of mitochondrial biogenesis and are necessary for renal recovery [33]. As shown in Figure 4B and C, overexpression of RacGAP1 up-regulated the mRNA expression levels of PGC-1α and NRF-2, along with the mtDNA copy numbers compared with control mice, confirming increased mitochondrial biogenesis. Western blot assay and IHC results showed increased PGC-1α expression in mice kidneys after pRacGAP1-LCP injection (Figure 4D–E). Mitochondrial morphology was evaluated in tubular cells by transmission electron microscopy. As shown in Figure 4F, many small and swollen mitochondria were observed in tubular cells of IRI mice, indicating mitochondria fragmentation. The mitochondria showed disordered cristae and cytoplasmic vacuolization, indicating impaired oxidative phosphorylation and ATP production. Notably, mitochondria were less damaged in pRacGAP1-LCP treated kidneys, with orderly packed cristae structure, less fragmentation, and fewer lipid droplets observed (Figure 4F).

**Figure 5:**
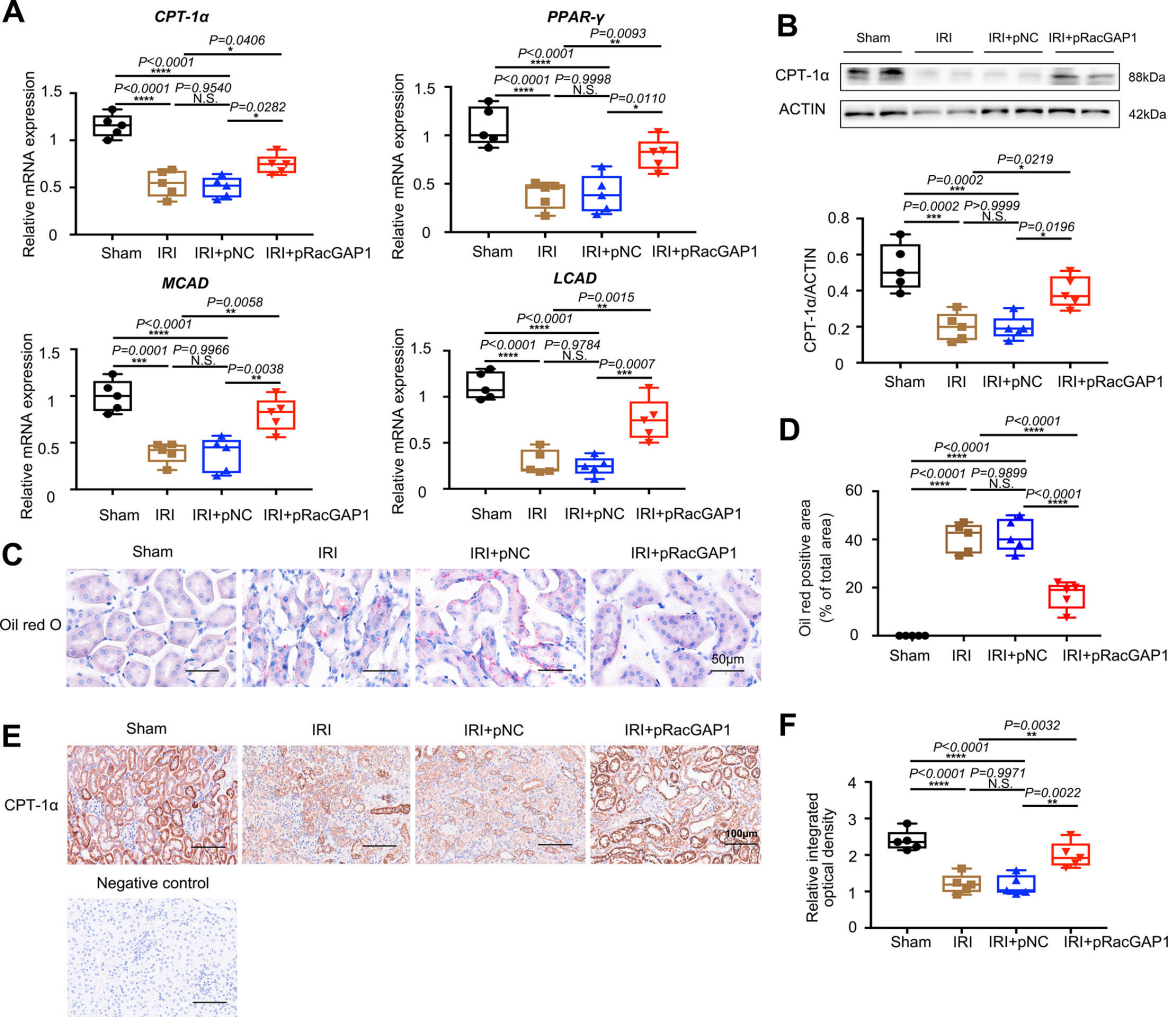
pRacGAP1-LCP protected against FAO defect in kidney tubules from IRI mice. (**A**) Real-time PCR analysis showing the mRNA abundance of CPT-1α, PPAR-γ, MCAD, and LCAD (*n* = 5). Significance was analyzed using one-way ANOVA followed by Tukey’s test. (**B**) Western blot assay showing the protein abundance of CPT-1α (*n* = 5). Significance was analyzed using one-way ANOVA followed by Tukey’s tests. (**C-D**) Oil Red O staining for lipids in the kidney evaluating lipid accumulation (*n* = 5). Significance was analyzed using one-way ANOVA followed by Tukey’s tests. For each mouse, an average staining score was derived from ten random fields. Scale bar = 50 μm. (**E-F**) Representative micrographs showing anti-CPT-1α IHC staining (*n* = 5). For each mouse, an average staining score was derived from ten random fields. Significance was analyzed using one-way ANOVA followed by Tukey’s test. Ten random fields were taken from each kidney. Scale bar = 100 μm. Data were presented as box plots showing the median and interquartile range, with whiskers extending from the minimum to the maximum value. *P<0.05; **P<0.01; ***P<0.001; ****P<0.0001; N.S., no significance.

FAO is the main source of energy in renal proximal tubular epithelial cells and primarily occurs in the mitochondria [34]. We observed that RacGAP1 overexpression markedly increased renal CPT-1α, PPAR-γ, MCAD, and LCAD mRNA expressions, suggesting better FAO (Figure 5A). Consistent with these findings, RacGAP1 overexpression up-regulated the protein level of CPT-1α (Figure 5B). Lipid accumulation was evaluated by oil red O staining in kidney slices. Results showed that less positive staining occurred in RacGAP1-LCP treated mice (Figure 5C and D). IHC assay also demonstrated that increased CPT-1α expression in mice kidneys after pRacGAP1-LCP injection (Figure 5E and F). Together, these results uncovered that RacGAP1 overexpression alleviated IRI-induced mitochondria defects in tubular cells in mice kidneys.

### RacGAP1 enhanced mitochondrial homeostasis in mRTECs

Building on these findings, we conducted a comprehensive investigation into the role of RacGAP1 in regulating mitochondria homeostasis in mRTECs. We employed the ATP depletion–repletion (ATP D-R) model, which is commonly used *in vitro* model of renal IRI [31,35]. Briefly, cells were treated with CCCP (a mitochondrial uncoupler that inhibits ATP synthesis) to induce ATP depletion and then returned to a normal culture medium for ATP repletion as described previously [35].

**Figure 6:**
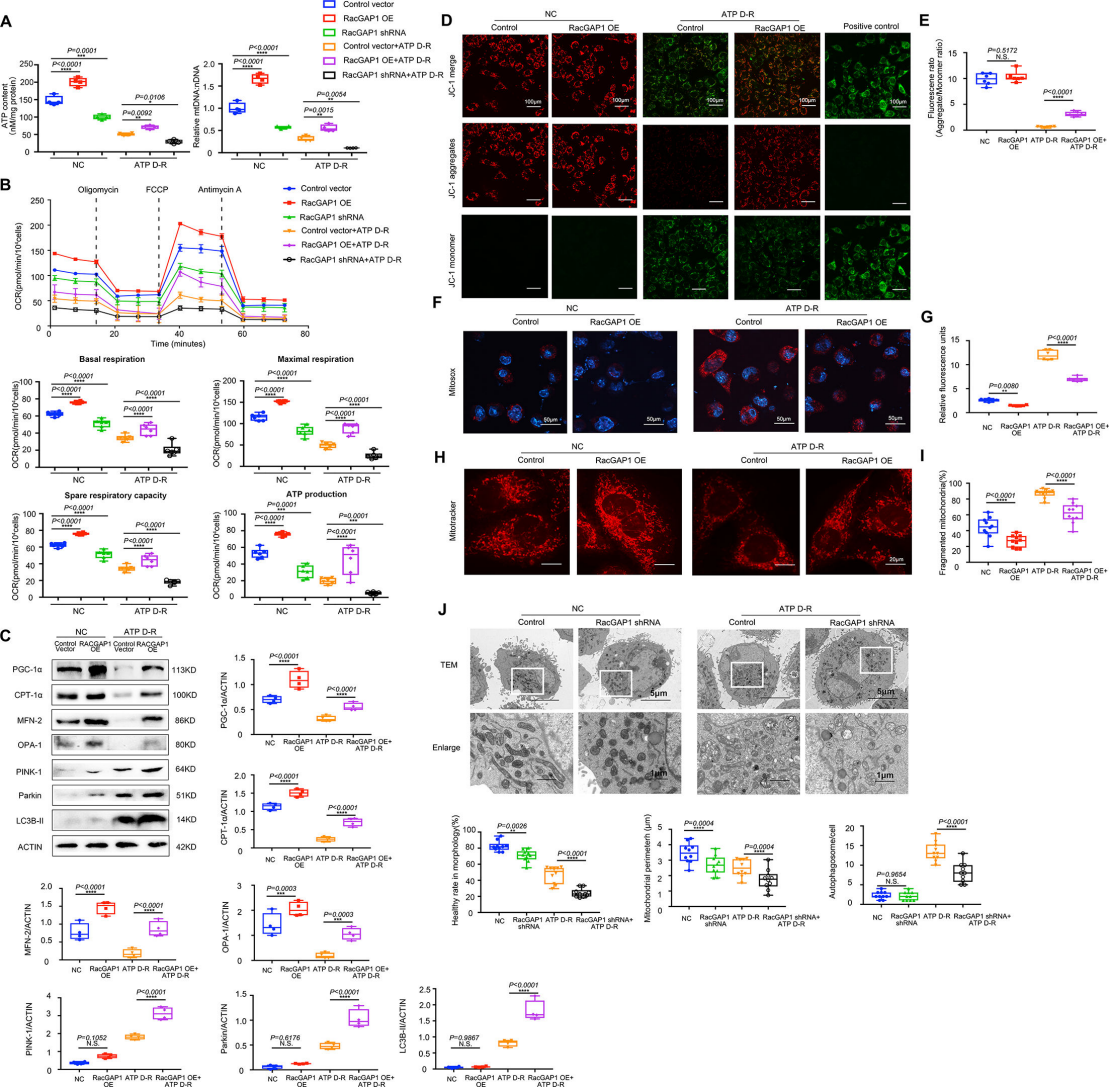
RacGAP1 promoted mitochondrial homeostasis in mRTECs after ATP D-R treatment. (**A**) Cellular ATP content and mtDNA copy numbers in mRTECs from different groups as indicated (*n* = 4). Significance was analyzed using two-way ANOVA followed by Sidak’s multiple comparisons test. (**B**) Oxygen consumption rate (OCR) measured by Seahorse assay. Maximal respiration, basal respiration, spare respiratory capacity, and ATP production were assessed under both basal and ATP D-R conditions (*n* = 6). Significance was analyzed using two-way ANOVA. (**C**) Western blot assay analysis of PGC-1α, CPT-1α, MFN-2, OPA-1, PINK-1, Parkin, LC3B-II protein levels in mRTECs with or without RacGAP1 overexpression under both basal and ATP D-R conditions (*n* = 4). Significance was analyzed using two-way ANOVA followed by Sidak’s multiple comparisons test. (**D**) Representative images of JC-1 staining. Scale bar = 100 μm. (**E**) Quantitative analyses of JC-1 fluorescence intensity (red/green) using the wavelength method (*n* = 6). Significance was analyzed using two-way ANOVA followed by Sidak’s multiple comparisons test. (**F**) Representative images of MitoSOX staining. Scale bar = 50 μm. (**G**) Quantitative analyses of MitoSOX staining using the wavelength method (*n* = 6). Significance was analyzed using two-way ANOVA followed by Sidak’s multiple comparisons test. (**H**) Representative images of MitoTracker staining. Scale bar = 20 μm. (**I**) Quantitative analyses of the percent of fragmented mitochondria using ImageJ software. Ten cells were randomly selected from each group to observe the mitochondrial morphology. Significance was analyzed using two-way ANOVA. (**J**) Representative transmission electron micrographs in control or RacGAP1-knockdown cells with or without ATP D-R treatment. Low-magnification image, scale bar = 5 μm. High-magnification image, scale bar = 1 μm. Ten cells were randomly selected from each group to observe the mitochondrial morphology. The healthy mitochondrial percent, the mitochondrial perimeter and the number of autophagosomes were calculated. Significance was analyzed using two-way ANOVA followed by Sidak’s multiple comparisons test. Data were presented as box plots showing the median and interquartile range, with whiskers extending from the minimum to the maximum value. *P<0.05; **P<0.01; ***P<0.001; ****P<0.0001; N.S., no significance.

Exposure to ATP D-R significantly impaired mitochondrial function in mRTEC cells, as evidenced by reduced ATP content and mtDNA copy numbers. These adverse effects were markedly attenuated by RacGAP1 overexpression. Knockdown of RacGAP1 exerted converse effects on mitochondrial function and caused a more severe decrease in ATP production and mtDNA copy numbers (Figure 6A). Then, we examined the OCR using the seahorse assay. Under both control and ATP D-R conditions, RacGAP1 overexpression increased maximal respiratory capacity, maximal respiration, spare respiratory capacity, and ATP production in mRTECs. Conversely, RacGAP1 knockdown impaired these respiratory parameters compared with the control group (Figure 6B).

Consistent with the improved respiratory function, RacGAP1 overexpression enhanced mitochondria biogenesis and FAO under both control and ATP D-R conditions, as shown by up-regulation of PGC-1α and CPT-1α. RacGAP1 overexpression also enhanced the mitochondrial fusion-related proteins (MFN2 and OPA1) under both conditions. Notably, RacGAP1 overexpression elevated the expression of mitophagy-related proteins (PINK1 and Parkin) along with the microtubule-associated protein 1 light chain 3B-II (LC3B-II) only in the ATP D-R condition (Figure 6C).

Under the control condition, RacGAP1 overexpression did not significantly alter the mitochondrial membrane potential, as indicated by strong red JC-1 fluorescence in both groups. ATP D-R treatment led to a drastic decrease in red JC-1 fluorescence in mRTECs with cells displaying intense green monomer fluorescence. RacGAP1 overexpression significantly attenuated this depolarization, as shown by increased red fluorescence after ATP D-R treatment (Figure 6D–E). RacGAP1 reduced mitochondrial ROS levels under both conditions, as measured by the MitoSOX assay (Figure 6F–G). The MitoTracker assay demonstrated a decrease in the percentage of cells with mitochondria in a fragmented state in the RacGAP1 overexpression group under both conditions (Figure 6H–I).

Transmission electron microscopy further confirmed the protective role of RacGAP1. RacGAP1-knockdown cells exhibited a lower proportion of healthy mitochondria under both conditions, suggesting that RacGAP1 was essential for preserving mitochondrial morphology. Mitochondria were smaller and more circular in RacGAP1-knockdown cells, indicating that RacGAP1 prevented mitochondrial fragmentation. Moreover, following ATP D-R treatment, RacGAP1 knockdown reduced the number of autophagosomes (Figure 6J).

Together, these findings support the conclusion that RacGAP1 contributes to mitochondrial homeostasis by enhancing mitochondrial biogenesis, promoting mitophagy and mitochondrial fusion, enhancing FAO, and attenuating oxidative stress.

## Discussion

**Figure 7:**
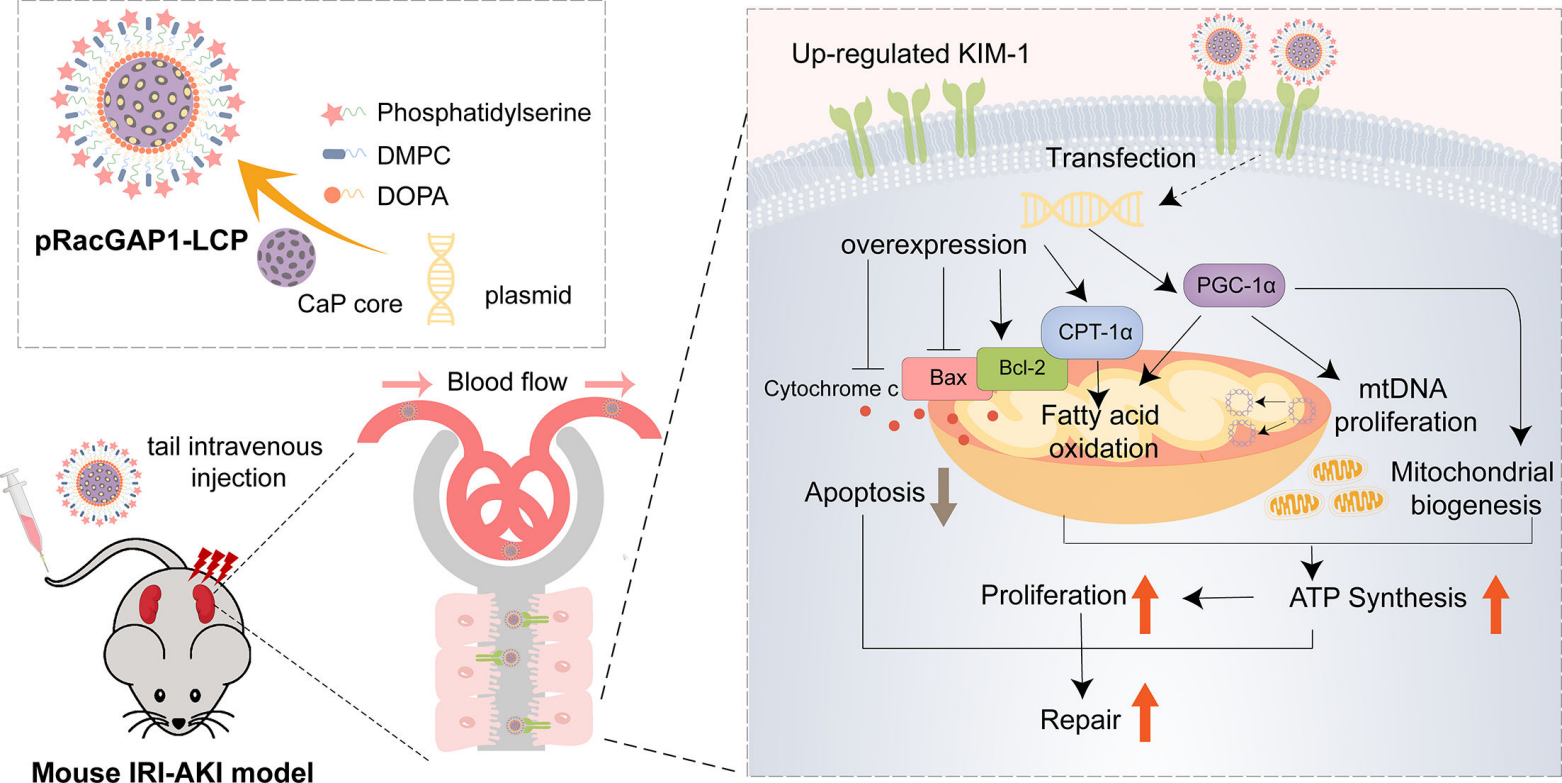
An illustration of the synthesis of pRacGAP1-LCP and its influences on renal IRI. RacGAP1 overexpression plasmids were encapsulated in the calcium phosphate cores. Then the pRacGAP1-loaded calcium phosphate cores were coated with lipids and phosphatidylserine to prepare the pRacGAP1-LCP nanoparticles. After intravenous administration, pRacGAP1-LCP accumulated in the kidney through blood circulation. RacGAP1 overexpression alleviated apoptosis and improved mitochondrial homeostasis, thus facilitating renal repair.

This study verified an approach to manufacture pRacGAP1-LCP in the treatment of ischemic AKI. As illustrated in Figure 7, delivering RacGAP1 plasmids by LCP nanoparticles enhanced its targeting ability to the injured kidney and exerted notable reno-protective effects by promoting mitochondrial homeostasis in mRTECs. Our findings suggest that pRacGAP1-LCP constitutes an effective nanotherapeutic for the treatment of AKI.

RacGAP1 catalyzes Rho GTPase and facilitates the translation between the GDP-bound inactive state and the GTP-bound active state [36]. As the component of the central spindle complex, RacGAP1 has been well studied within cytokinesis [37–39]. Exit of nuclear RacGAP1 to the cytoplasm activates RhoA and promotes cell detachment [40]. Studies have reported that RacGAP1 expression is up-regulated in various malignancies, exerting pro-oncogenic effects independent of its GTPase activity [9,41,42]. RacGAP1 is essential for cell survival and proliferation across diverse cell types, such as hepatocellular carcinoma cells [43,44], uterine carcinosarcoma cells [45], pseudostratified epithelial cells [46] and hematopoietic cells [47]. Previous studies on RacGAP1 mainly focused on the role of RacGAP1 in regulating cytoplasmic division and promoting tumor progression. In our previous study, overexpression of RacGAP1 significantly thwarted apoptosis and promoted repair in renal tubular cells *in vitro* [13]. The sequencing results of single-cell transcriptomes from both the unilateral IRI model and the unilateral ureteral obstruction (UUO) model (http://humphreyslab.com/SingleCell/) reveal that RacGAP1 is significantly up-regulated in the benignly proliferating and repairing PTECs (S upplementary F igure S 1). Our study further showed that overexpression of RacGAP1 during IRI reduced renal histological injury *in vivo.* We also originally elucidated *in vivo* and *in vitro* that RacGAP1 promoted mitochondrial homeostasis in PTECs.

One of the most important pathological features of AKI is ATP depletion in PTECs and lack of energy supply directly leads to cell dysfunction and apoptosis [48]. Activation of mitochondrial biosynthesis during AKI can increase numbers of mitochondria and enhance aerobic respiration capacity, thus promoting kidney repair. Recent studies have focused on improving the mitochondrial function to ameliorate AKI [35,49,50]. Our data revealed that RacGAP1 overexpression not only enhanced mitochondrial biogenesis and FAO but also elevated the protein levels of key mitophagy regulators and mitochondrial fusion regulators *in vitro*, suggesting a broader role for RacGAP1 in promoting mitochondrial homeostasis. Consistent with this, RacGAP1 overexpression induced a decrease in the percentage of cells with mitochondria in a fragmented state in MitoTracker assay. Electron microscopy images demonstrated a significant increase in damaged and fragmented mitochondria, as well as a decrease in the number of autophagosomes in RacGAP1-knockdown cells. However, it is important to emphasize that while our data provide preliminary evidence suggesting potential effects of RacGAP1 on mitophagy and mitochondrial dynamics, these observations remain exploratory. The precise regulatory mechanisms and functional significance of RacGAP1 in the clearance of damaged mitochondria and the balance of fusion and fission warrant further in-depth investigation.

Literature has illustrated that YAP, a transcriptional co-activator that regulates mitochondrial homeostasis by promoting both mitochondrial biogenesis and mitophagy [51]. We have previously reported that RacGAP1 exerted its function on cell proliferation and apoptosis in tubular cells through activation of YAP [13]. While our current data have shown a protective role for RacGAP1 in mitochondrial quality control, whether the effect was mediated through the activation of YAP needs to be elucidated in our future research. Ren et al. also reported that RacGAP1 promoted mtDNA synthesis by up-regulating the expression of DNA Methyltransferase 1 (DNMT1) [14]. DNMT1 inhibits apoptosis of renal tubular epithelium and plays a protective role in the cisplatin-induced AKI model [52]. Whether RacGAP1 regulates DNMT1 during AKI remains to be clarified.

Nanoparticles have become a powerful tool in the diagnosis and treatment of many diseases, but the current research on the use of nanoparticles in AKI is still in its infancy. Previous studies have shown that calcium phosphate nanoparticles as a plasmid carrier have good safety and stability in cancer therapy [17,53]. In this study, pRacGAP1-LCP was constructed, and phosphatidylserine that could specifically bind to KIM-1 was connected to elevate its renal-targeting ability. We confirmed that pRacGAP1-LCP had a high transfection efficiency and achieved high expression of RacGAP1 in mice kidney tissues. The nanoparticles successfully overcame the delivery problems of traditional nucleic acid drugs, such as easy degradation, short circulation time, and difficulty in passing the membrane barrier. This study provides a new design idea and experimental basis for AKI precision therapy and has the prospect of further application.

Although significant accumulation of nanoparticles was observed in the liver and spleen, H&E staining revealed no apparent tissue toxicity or signs of altered proliferation in these organs compared with IRI-treated controls (Supplementary Figure S2). We speculate that the dominant uptake may be by non-parenchymal cells like Kupffer cells in the liver and macrophages in the spleen, where the biological function of RacGAP1 might not directly translate to driving proliferation in the same manner as in cancer cells. Nonetheless, the long-term biodistribution and potential off-target effects remain an important consideration for clinical translation.

This study also has some shortcomings. Whether RacGAP1 is highly expressed in renal biopsy tissues of AKI patients, and the correlation between RacGAP1 expression level and blood creatinine and urea nitrogen levels of patients need to be further clarified. At the same time, whether RacGAP1 knockdown in mice kidneys aggravates AKI remains to be determined. It has been reported that RacGAP1 knockout mice died early in development due to mitotic defects, suggesting that RacGAP1 is essential for survival [54]. Conditional knockout mice needed to be constructed to further verify the effect of RacGAP1 inhibition on AKI. In addition, we used the IRI model of mice with 21 min of bilateral renal ischemia followed by 72 h of reperfusion. In this model, the creatinine of mice has returned to normal levels 72 h after reperfusion. The role of RacGAP1 in more severe IRI models and other AKI models remains to be further studied. Another limitation is the short-term nature of our toxicological analysis. Although our histological analysis revealed no apparent damage in liver and spleen (Supplementary Figure S2), these assessments were conducted over a relatively short-term period. Consequently, the potential for long-term adverse effects cannot be entirely excluded based on the present data. Future work will focus on longitudinal studies to monitor the long-term biocompatibility and chronic toxicity of the treatment.

### Conclusions

LCP nanoparticles carrying RacGAP1 overexpression plasmids have been developed to alleviate renal IRI in mice in the present study. The nanoparticles stay stable during delivery in blood circulation, accumulate in the IRI kidney, and successfully elevate the expression of RacGAP1. This study shows for the first time that RacGAP1 protects against IRI by improving mitochondrial homeostasis. This biomimetic nanoparticle drug delivery system may provide an attractive strategy for the prevention and treatment of AKI in clinical practice.

**Clinical Perspectives**

- Our previous study revealed that RacGAP1 plays a crucial role in promoting repair of tubular epithelial cells in vitro. However, the therapeutic strategies to enhance RacGAP1 expression *in vivo* following acute kidney injury (AKI) remain to be explored.
- Notably, lipid-coated calcium phosphate nanoparticles encapsulating RacGAP1-overexpressing plasmids effectively attenuated ischemia/reperfusion injury (IRI)-induced renal tubular damage, apoptotic cell death, and inflammatory responses. Mechanistic analyses revealed that RacGAP1 exerted its reno-protective effects by enhancing mitochondrial biogenesis and fatty acid oxidation, thereby maintaining mitochondrial homeostasis.
- These findings identify RacGAP1 as a novel therapeutic target for ischemic AKI, with the potential to improve renal recovery. Moreover, this study demonstrates the feasibility of nanoparticle-mediated plasmid delivery for AKI treatment in preclinical models, providing a novel therapeutic avenue for AKI.

## Supplementary material

online supplementary figure 1

online supplementary figure 2

online supplementary material 1

## Data Availability

The data supporting the findings of this study could be obtained from the corresponding author.

## Open Access

This article has been published open access under our Subscribe to Open programme, made possible through the support of our subscribing institutions, learn more here: https://portlandpress.com/pages/open_access_options_and_prices#conditional

## Abbreviations

AKI: acute kidney injury
IRI: ischemia/reperfusion injury
LCP: lipid-coated calcium phosphate
RacGAP1: Rac GTPase-activating protein 1
mRTECs: mouse renal tubular epithelial cells
