# Supplementary figures and images for: Nanoparticle-mediated overexpression of RacGAP1 protects against renal ischemia/reperfusion injury by maintaining mitochondrial homeostasis
Source: Clin Sci (Lond). 2025 Nov 27;139(22):1571–90. doi: 10.1042/CS20256110 (PMC12751063; doi:10.1042/CS20256110)

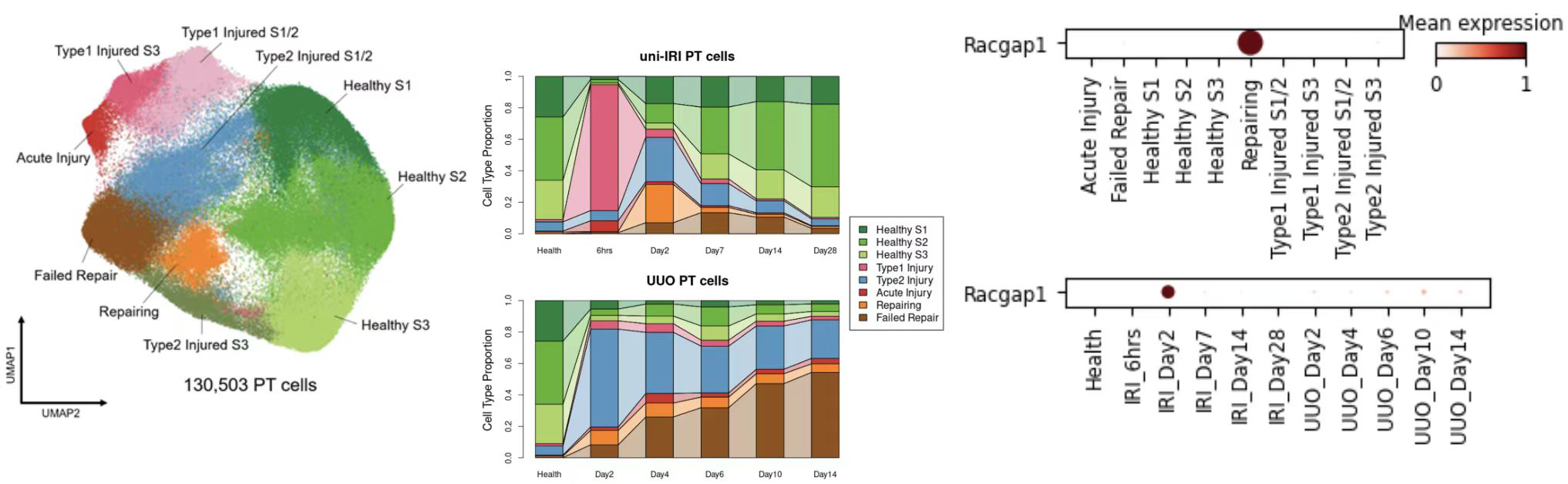

Supplement: online supplementary figure 1 [file CS-139-22-CS20256110-s001.tif]

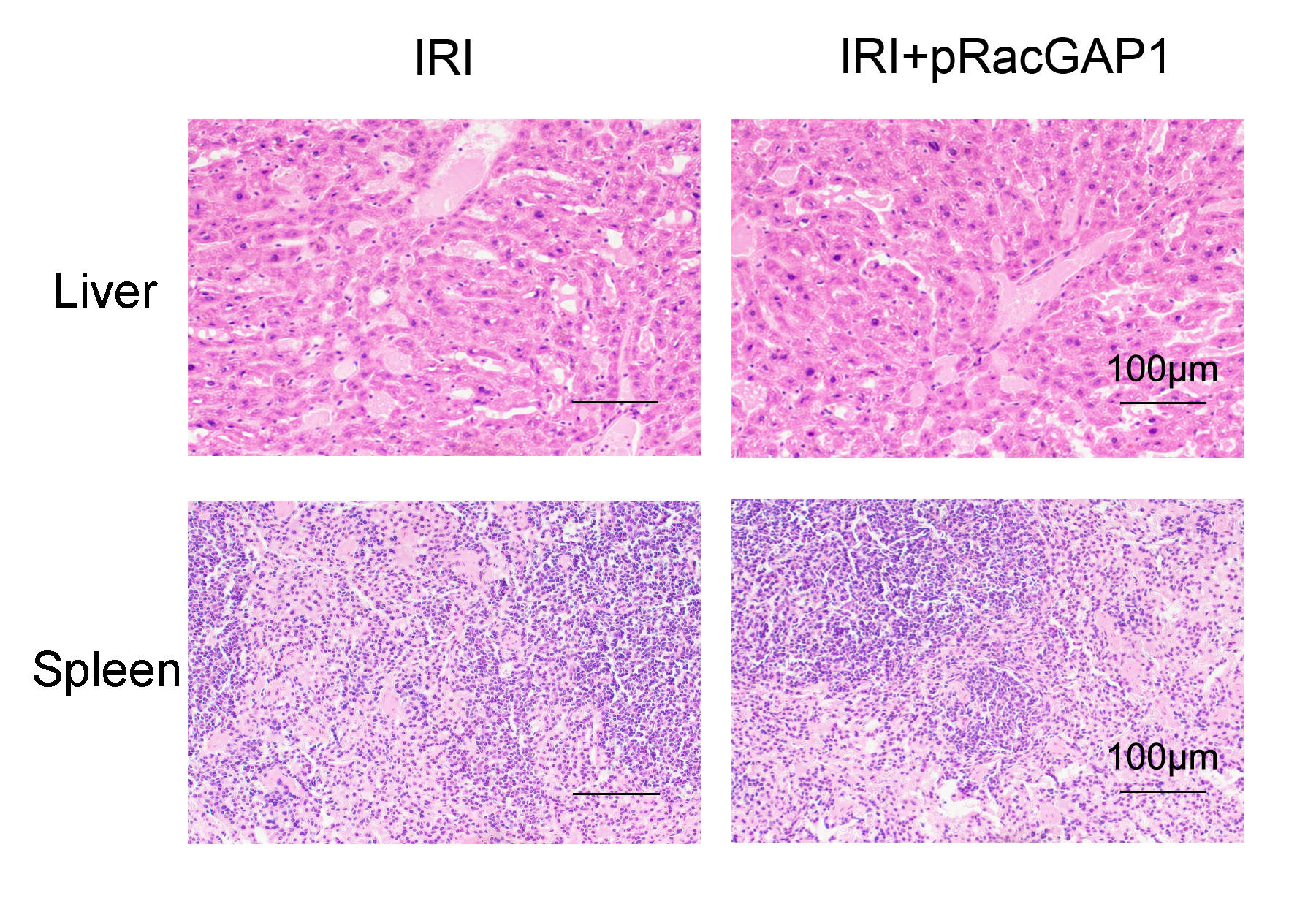

Supplement: online supplementary figure 2 [file CS-139-22-CS20256110-s002.tif]

FIGURE 1

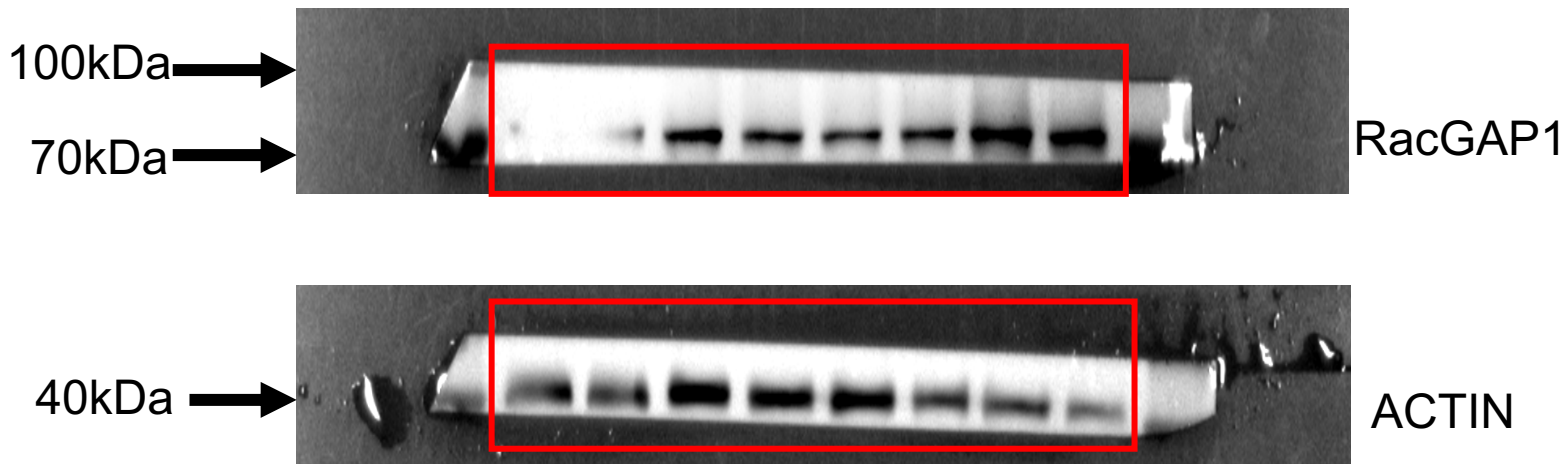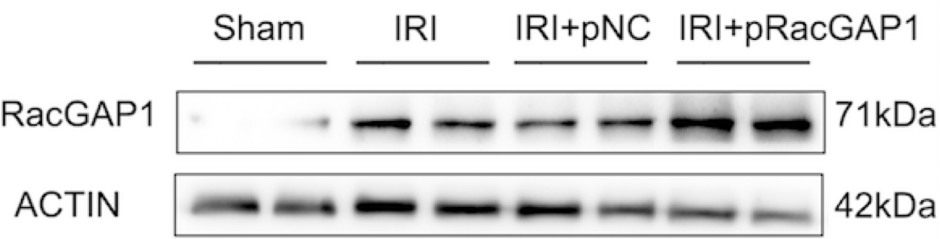

FIGURE 2

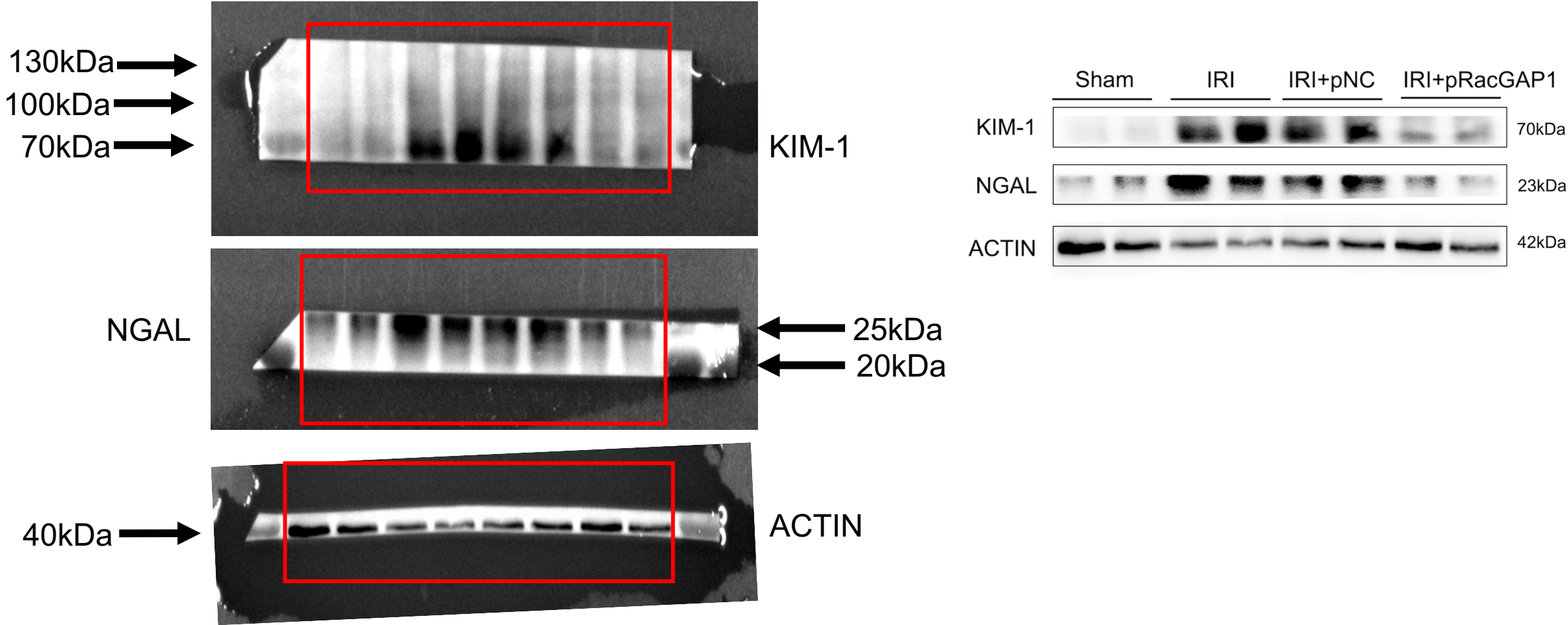

FIGURE 3

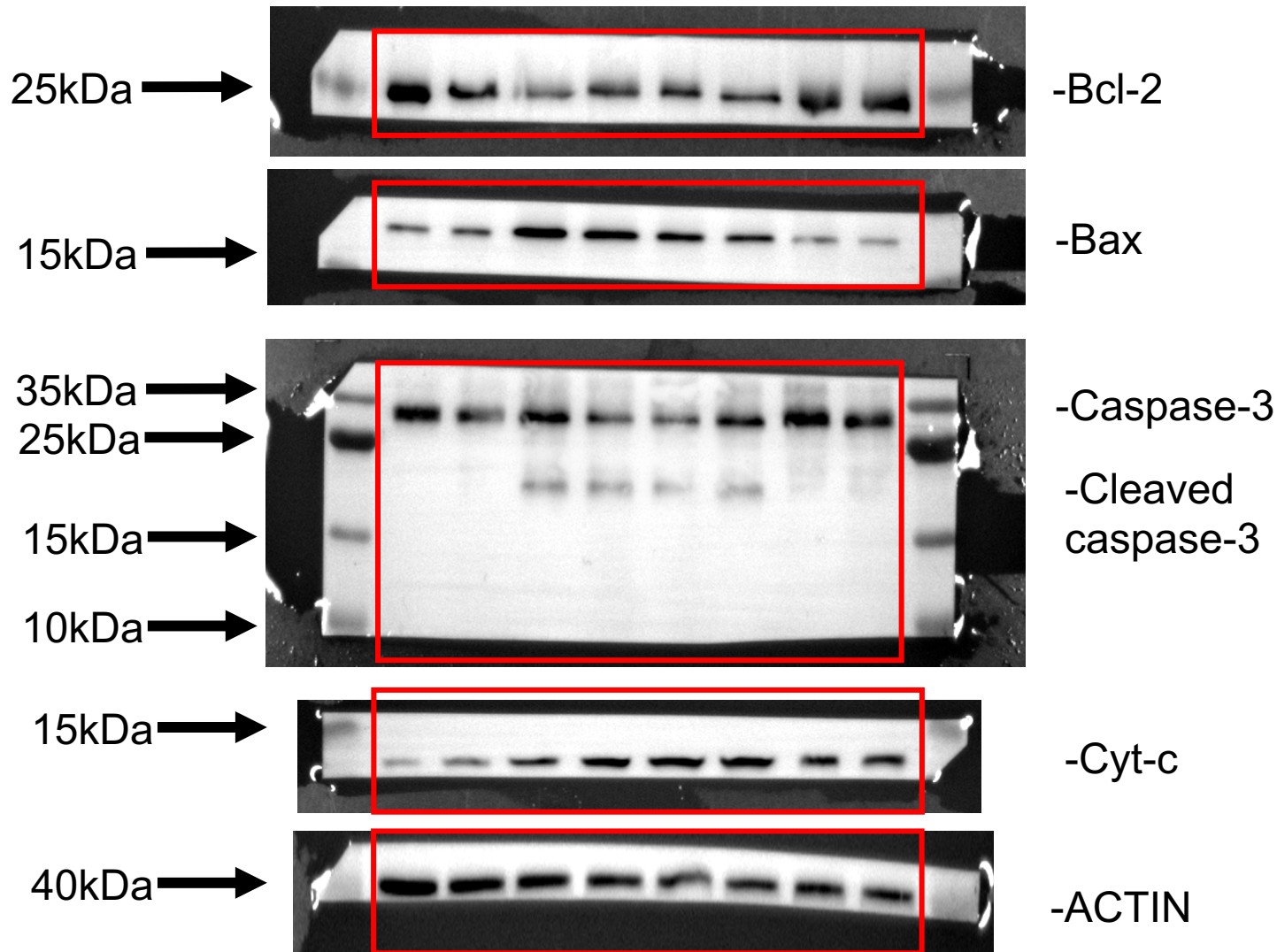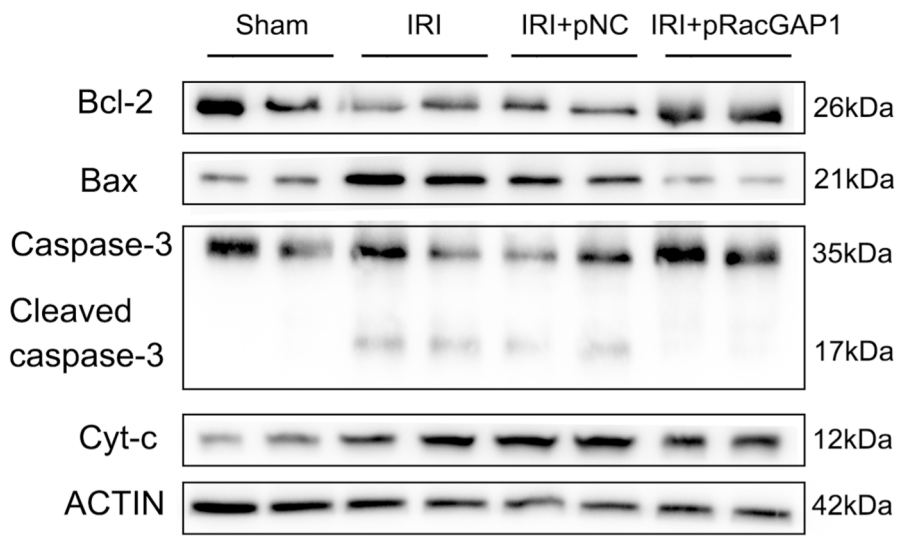

FIGURE 4

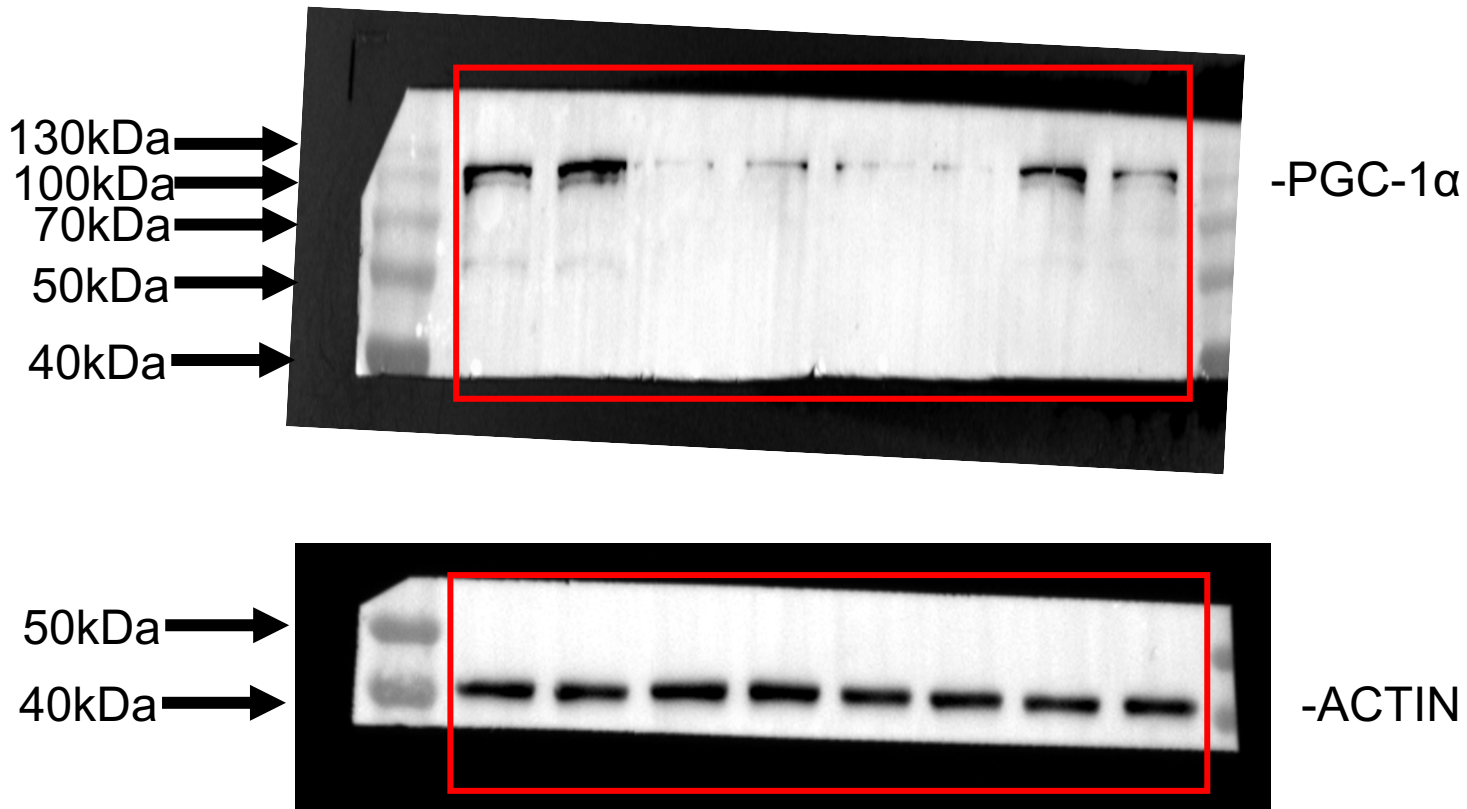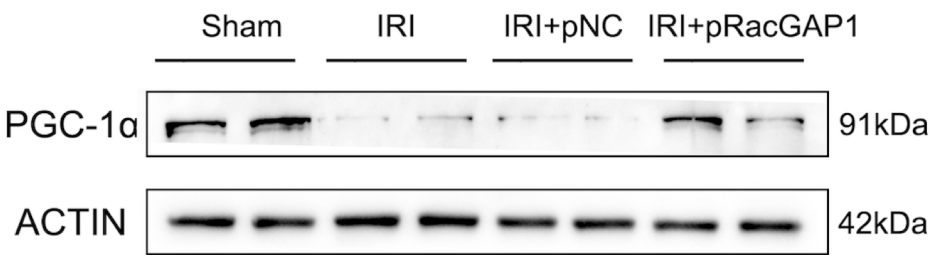

FIGURE 5

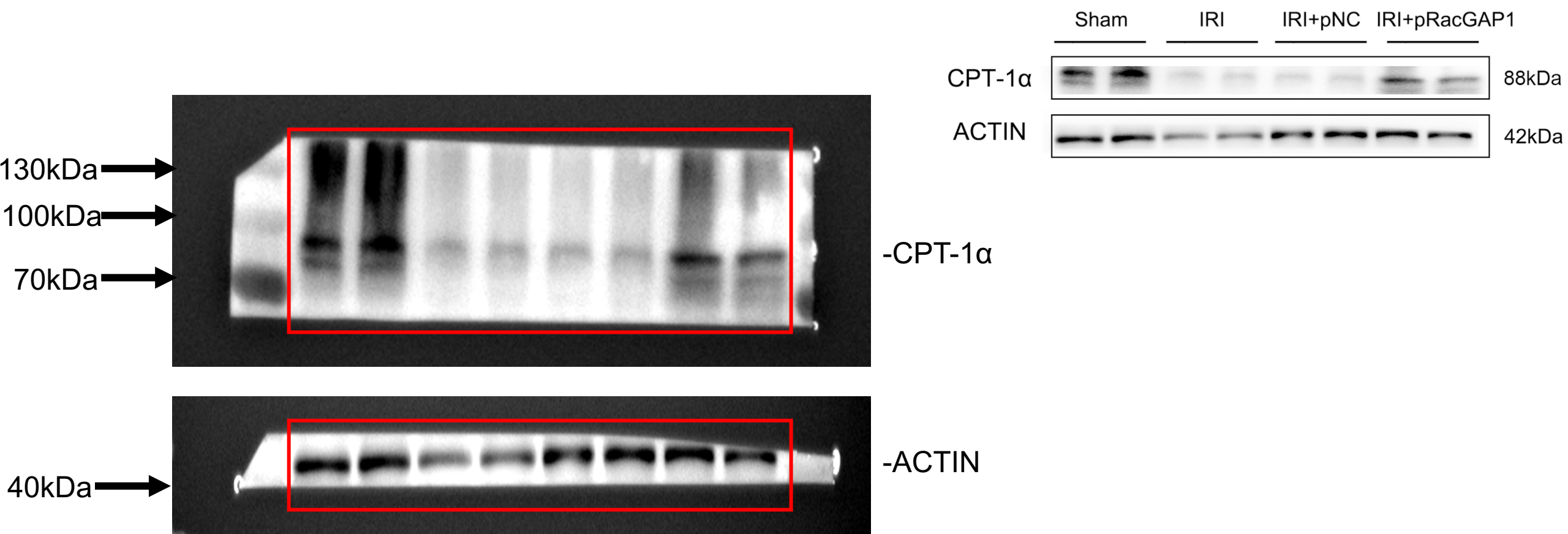

FIGURE6

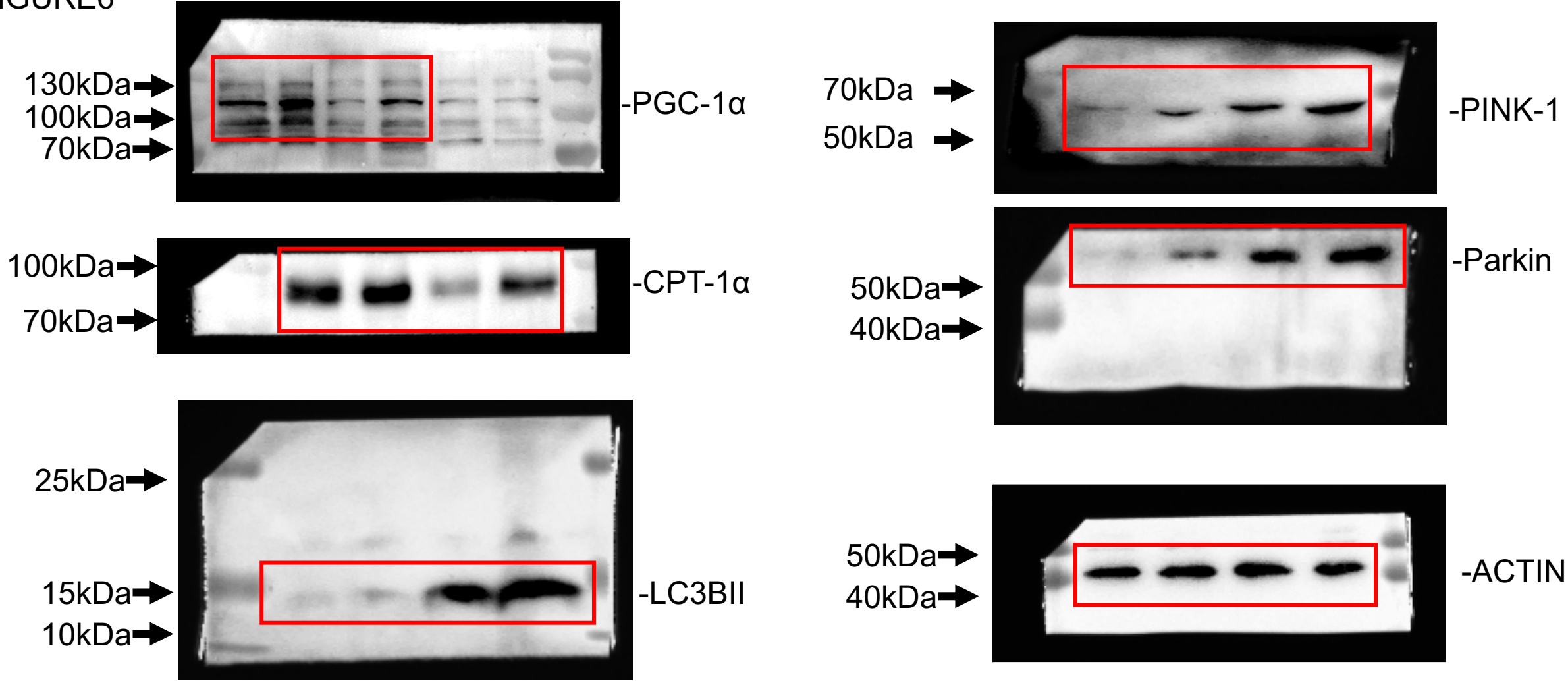

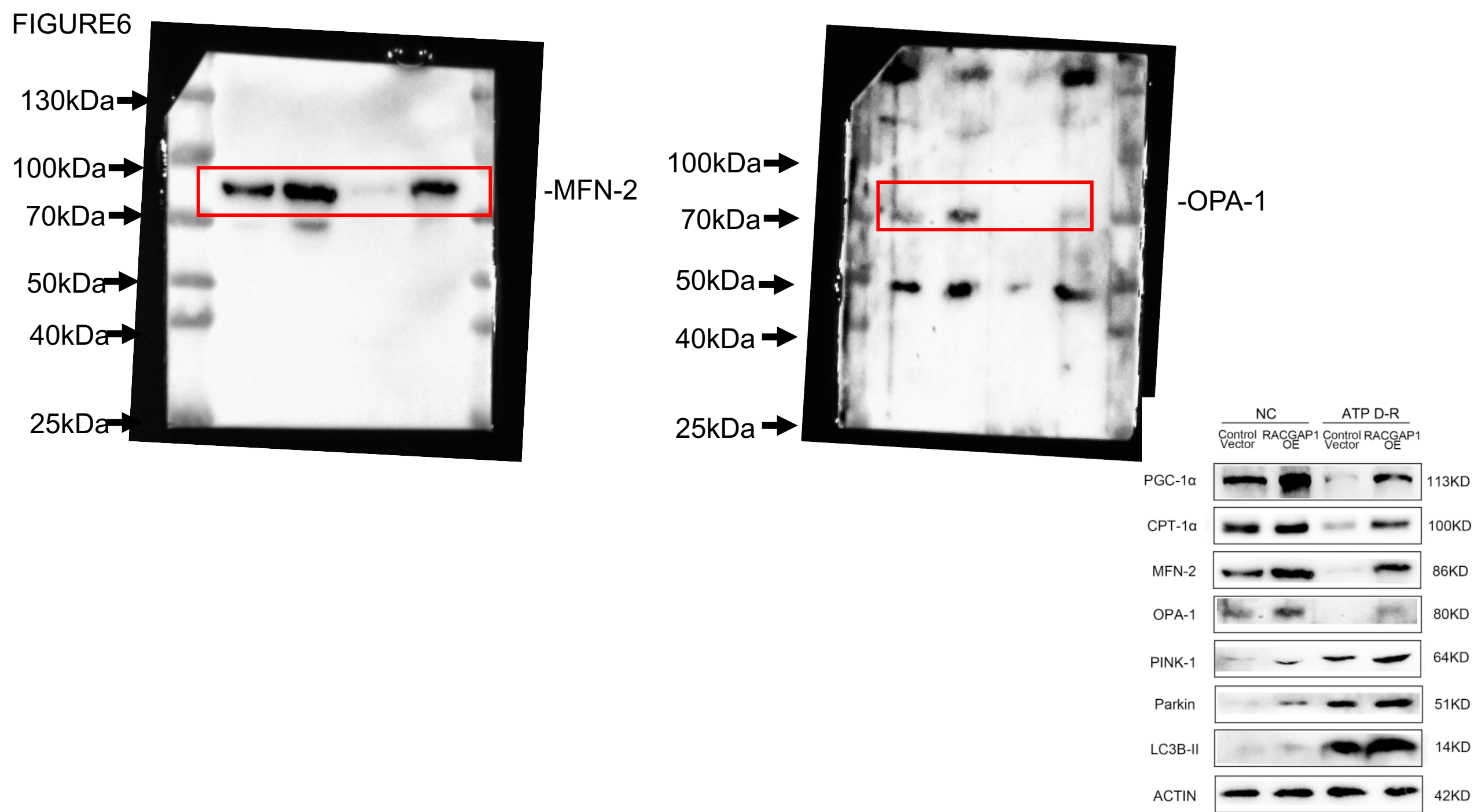

Supplement: online supplementary material 1 [file CS-139-22-CS20256110-s003.pdf]
